# Easily Accessible and Solution‐Stable Ni(0) Precatalysts for High‐Throughput Experimentation

**DOI:** 10.1002/chem.202403960

**Published:** 2025-01-28

**Authors:** Gilian T. Thomas, Odhran D. Cruise, Daelin Peel‐Smith, Nahiane Pipaón Fernández, Charles Killeen, David C. Leitch

**Affiliations:** ^1^ Department of Chemistry University of Victoria 3800 Finnerty Rd. Victoria BC V8P 5 C2 Canada

**Keywords:** nickel precatalyst, homogenous catalysis, cross-coupling, high-throughput experimentation, organic synthesis

## Abstract

We report the synthesis, characterization, and catalytic applications of *N*,*N’*‐diaryl diazabutadiene (DAB) Ni(0) complexes stabilized by alkene ligands. These complexes are soluble and stable in several organic solvents, making them ideal candidates for *in situ* catalyst formation during high‐throughput experimentation (HTE). We used HTE to evaluate these Ni(0) precatalysts in a variety of Suzuki and C−N coupling reactions, and they were found to have equal or better performance than the still‐standard Ni(0) source, Ni(COD)_2_.

## Graphical abstract

Several α‐diimine stabilized Ni(0) complexes are active precursors for *in situ* catalyst formation, with particular applicability to high‐throughput experimentation. These compounds are easily prepared and solution stable, and function well in several cross‐coupling reactions.

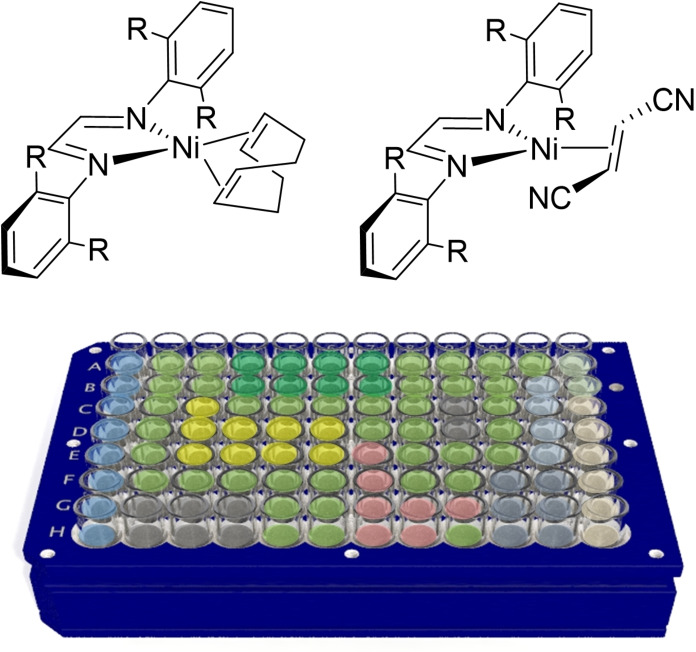

## Introduction

The urgent need for earth abundant metal catalysts to replace the commonly used precious metal catalysts is a focal issue in achieving sustainable chemical synthesis.[^1^, ^2^, ^3^, ^4^, ^5^] In particular, replacing Pd‐based catalyst systems with those based on Ni is an attractive solution to the known issues associated with Pd, including its volatile cost and availability.[^1^, ^6^, ^7^, ^8^] These issues become significantly more prominent when considering large‐scale chemistry, such as for pharmaceutical or agrochemical manufacturing. Unfortunately, one of the most common (and successful) sources of Ni(0), Ni(COD)_2_ (COD=1,5‐cyclooctadiene), is notoriously air‐sensitive and thermally unstable, making prolonged storage a major challenge.^[9]^ Furthermore, despite being earth abundant, it is relatively costly, which can be a barrier to its use in manufacturing.[^10^, ^11^]

Currently, there is a dearth of well‐defined Ni(0) or Ni(II) precursors suitable for microscale high‐throughput experimentation (HTE). While significant strides have been made in Ni precatalyst development,[^12^, ^13^, ^14^, ^15^, ^16^] many of the Ni(0) variants remain poorly soluble/insoluble and/or unstable in common HTE‐compatible solvents, making homogeneous reaction conditions difficult to achieve and the use of stock solutions not feasible. Comparatively, commercially available Ni(II) salts (e. g. NiBr_2_(dme) or Ni(acac)_2_) require a reducing agent to form the Ni(0) species *in situ*, potentially introducing hidden factors and robustness concerns into screening campaigns.^[17]^

**Figure 1:**
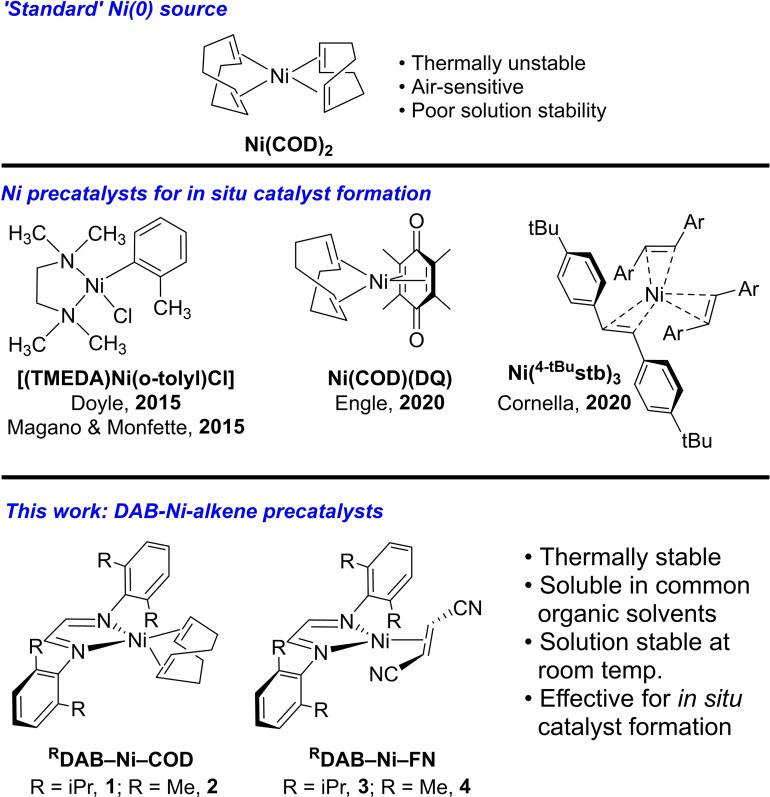
Ni complexes used for *in situ* catalyst formation in cross‐coupling reactions, including Ni(COD)_2_, Ni(COD)(DQ),^[15]^ [(TMEDA)Ni(*o*‐tolyl)Cl],[^18^, ^19^] Ni(^4−tBu^stb)_3_,^[21]^ and DAB‐Ni‐alkene complexes **1**–**4** studied herein.

There are several relevant recent examples of stable Ni precursors suitable for *in situ* catalyst formation (Figure 1), including Ni(COD)(DQ),^[15]^ [(TMEDA)Ni(*o*‐tolyl)Cl],[^18^, ^19^] and diarylstilbene‐stabilized Ni(0) complexes, with Ni(^4‐tBu^stb)_3_ as an exemplar,[^20^, ^21^, ^22^] among others.^[12]^ The subsequent report of a library of air‐stable Ni(0) precursors by Engle *et al*. emphasizes the need for precatalyst diversity to achieve desired reactivity across different reaction classes.^[16]^

**Figure 2:**
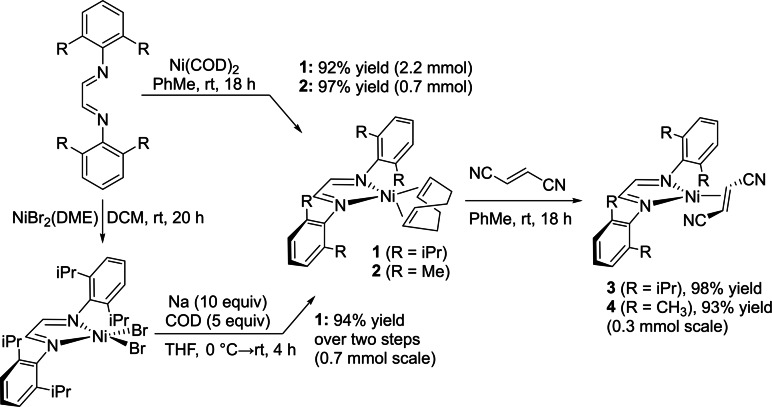
Synthesis of **1**–**4** starting from either Ni(COD)_2_ or NiBr_2_(DME) (**1**).

Herein, we disclose several HTE‐compatible Ni(0) precatalysts with demonstrated reactivity in multiple cross‐coupling reaction classes (Figure 1, **1**–**4**). Stemming from our previous work using diazabutadiene (DAB) ligands to generate stable and active Pd(0) precatalysts,^[23]^ we adopted a similar design for Ni. In addition to the *N,N’*‐bis(2,6‐dimethylphenyl) diazabutadiene (^DMP^DAB) ligand, we also evaluated the more sterically encumbered *N,N’*‐bis(2,6‐diisopropylphenyl) diazabutadiene (^DIPP^DAB) analogue. These precatalysts can be easily prepared by treating a solution of Ni(COD)_2_ in toluene with the desired DAB ligand, leading to a 95 % and 97 % yield of ^DIPP^DAB‐Ni‐COD (**1**) and ^DMP^DAB‐Ni‐COD (**2**), respectively (Figure 2).

## Results and Discussion

### Synthesis and Characterization of Ni(0) Complexes

Compound **1** was first reported in 1981 by Dieck *et al*., generated from a mixture of Ni(COD)_2_ and ^DIPP^DAB in diethyl ether followed by hexane extraction.^[24]^ In 1990, **1** was prepared from a mixture of tris(ethylene)Ni with ^DIPP^DAB in pentane.^[25]^ More recently, **1** was prepared in THF from Ni(COD)_2_ by Sgro and Stephan.^[26]^ In our studies, we switched to using toluene as the reaction solvent as the displaced COD byproduct was removed more efficiently *in vacuo* under these conditions, simplifying isolation. This synthetic method was also used to synthesize **2**, which is a new compound. No additional purification is required beyond simple filtration prior to evaporation, which is a key advantage of these precatalysts.

In light of the challenges associated with acquisition and storage of Ni(COD)_2_ (as well as its not insignificant cost from most suppliers), we have designed an alternative synthetic method to access **1** from NiBr_2_(DME). Metallation of the ^DIPP^DAB ligand to NiBr_2_(DME) in DCM leads to precipitation of (^DIPP^DAB)NiBr_2_. Following evaporation and hexane washes, reduction with Na^[24]^ in the presence of COD generates precatalyst **1** in 94 % yield (Figure 2).

While COD is a suitable stabilizing ligand for low‐valent Ni species, its chelating nature and potential for further reactivity make it a likely competitive inhibitor / substrate during catalysis. To access COD‐free Ni(0) precatalysts, we conducted ligand substitution reactions with an electron‐deficient alkene, fumaronitrile (FN), to produce novel complexes ^DIPP^DAB‐Ni‐FN (**3**) and ^DMP^DAB‐Ni‐FN (**4**) (Figure 2). The free COD released is easily removed *in vacuo* along with the solvent. Analogous DAB‐Pd‐FN and DAB‐Pt‐FN analogues have previously been prepared, though these are (to the best of our knowledge) the first Ni‐based examples.[^27^, ^28^] In both cases, the fumaronitrile ligands are not observable in the NMR spectra; however, we have confirmed their presence through IR spectroscopy and elemental analysis (see Supporting Information).

One of our primary goals in designing new catalyst precursors is to achieve physicochemical properties suitable for HTE array set‐up and execution.[^23^, ^29^, ^30^] This includes good solubility and room temperature solution stability of the complexes to enable solution‐based dispensing. We therefore evaluated the solubility of **1**–**4** (20 mg/mL initial charge) in benzene, toluene, and THF – key solvents for catalysis – as well as solubility of commercially available precursors Ni(COD)_2_ and Ni(COD)(DQ) (Table 1). For the DAB‐coordinated complexes, we observe >10 mg/mL solubility in every case, with complex **1** exhibiting at least 19 mg/mL solubility. While Ni(COD)_2_ also exhibits high solubility in these three solvents, we observe rapid (or instantaneous in the case of C_6_D_6_) decomposition to Ni black. Ni(COD)(DQ) is known to exhibit excellent stability, even in air; however, it has markedly lower solubility in these three solvents (4‐8 mg/mL).

**Table 1:** Solubility of Ni precatalysts in relevant solvents.^[a]^

Entry	Ni(0) Precatalyst	Solubility in C_6_D_6_ (mg/mL)	Solubility in d_8_‐PhMe (mg/mL)	Solubility in d_8_‐THF (mg/mL)
1	Ni(COD)_2_	Decomposed	>20	19
2	Ni(COD)(DQ)	8	4	6
3	**1**	>20	19	>20
4	**2**	>20	19	17
5	**3**	>20	17	11
6	**4**	>20	17	15

^[a]^ Measured using ^1^H NMR spectroscopy with 1,3,5‐trimethoxybenzene as internal standard; >20 mg/mL indicates the complex is at least this soluble.

**Figure 3:**
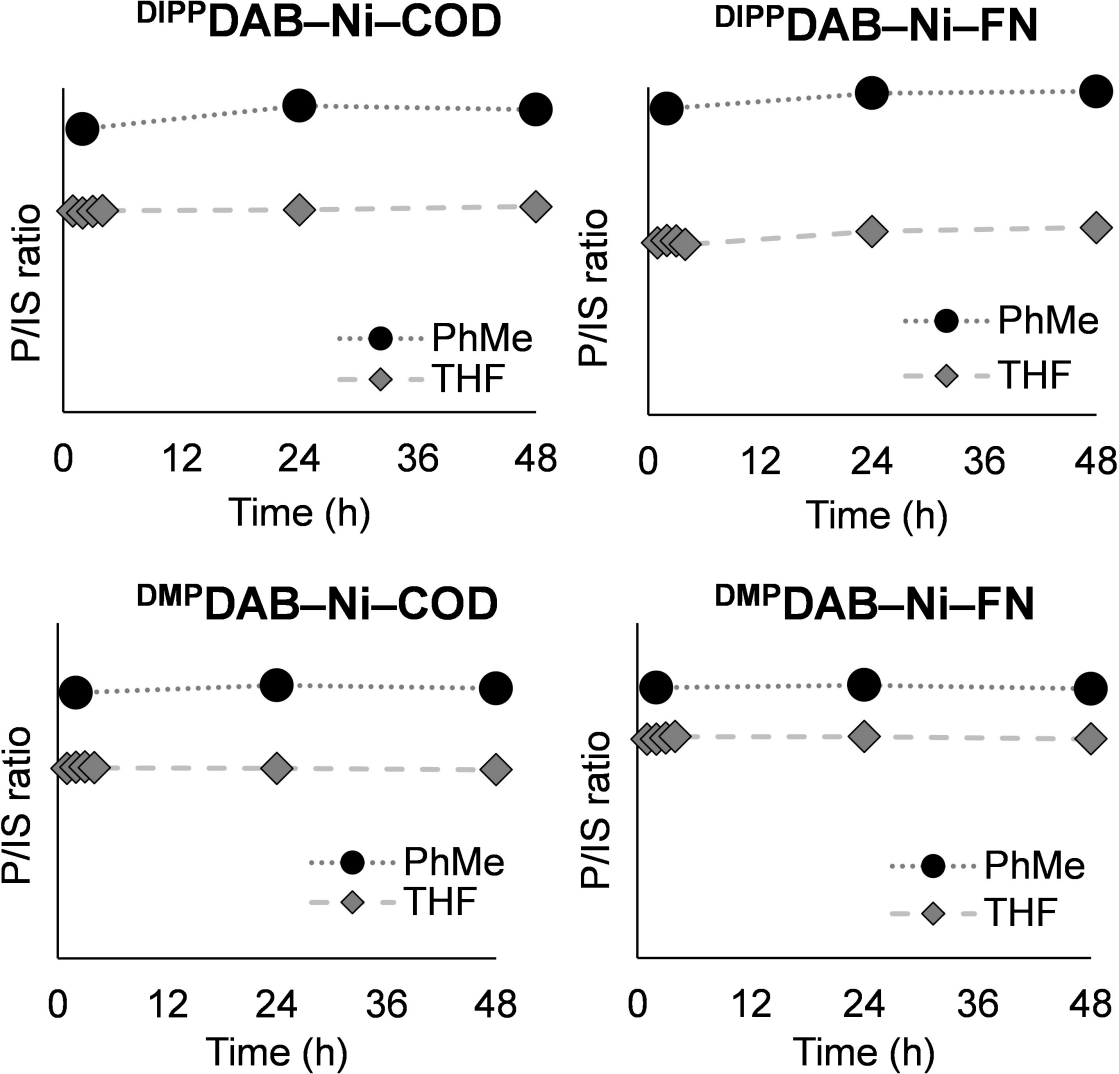
Solution stability of **1**–**4** determined by ^1^H NMR spectroscopy at room temperature in deuterated toluene and THF at maximum solubility (values from Table 1) under inert atmosphere. P/IS refers to peak area ratio between product (P) and internal standard (IS, 1,3,5‐trimethoxybenzene). See Supporting Information for more details.

From a solution stability standpoint, we monitored the concentration of **1**–**4** by ^1^H NMR spectroscopy over 48 hours in deuterated toluene and THF, using 1,3,5‐trimethoxybenzene as an internal standard. The initial concentration of the Ni complex is the maximum solubility from Table 1. In each case, the peak area ratio values are stable, and initial and final peak area ratios are within 10 % (Figure 3).

Furthermore, we observe no formation of free DAB or COD over a 48 hour time period. We also examined the stability of **1**–**4** in C_6_D_6_ (20 mg/mL) over a longer period, with excellent solution stability observed over 11 days (see Supporting Information for details). Accordingly, stock solutions of **1**–**4** can accurately and confidently be made and used in these solvents, providing HTE users with solution‐dispensing options. Finally, while **1**–**4** are air sensitive, we did test the air stability of **1** by exposing it to ambient atmosphere. Weighing **1** on the bench and then transferring it to the glovebox resulted in 86 % of **1** remaining intact, according to qNMR analysis using an internal standard (see SI for details).

### Catalytic Evaluation of DAB‐Supported Ni(0) Precatalysts for HTE and Larger‐Scale Applications

**Figure 4:**
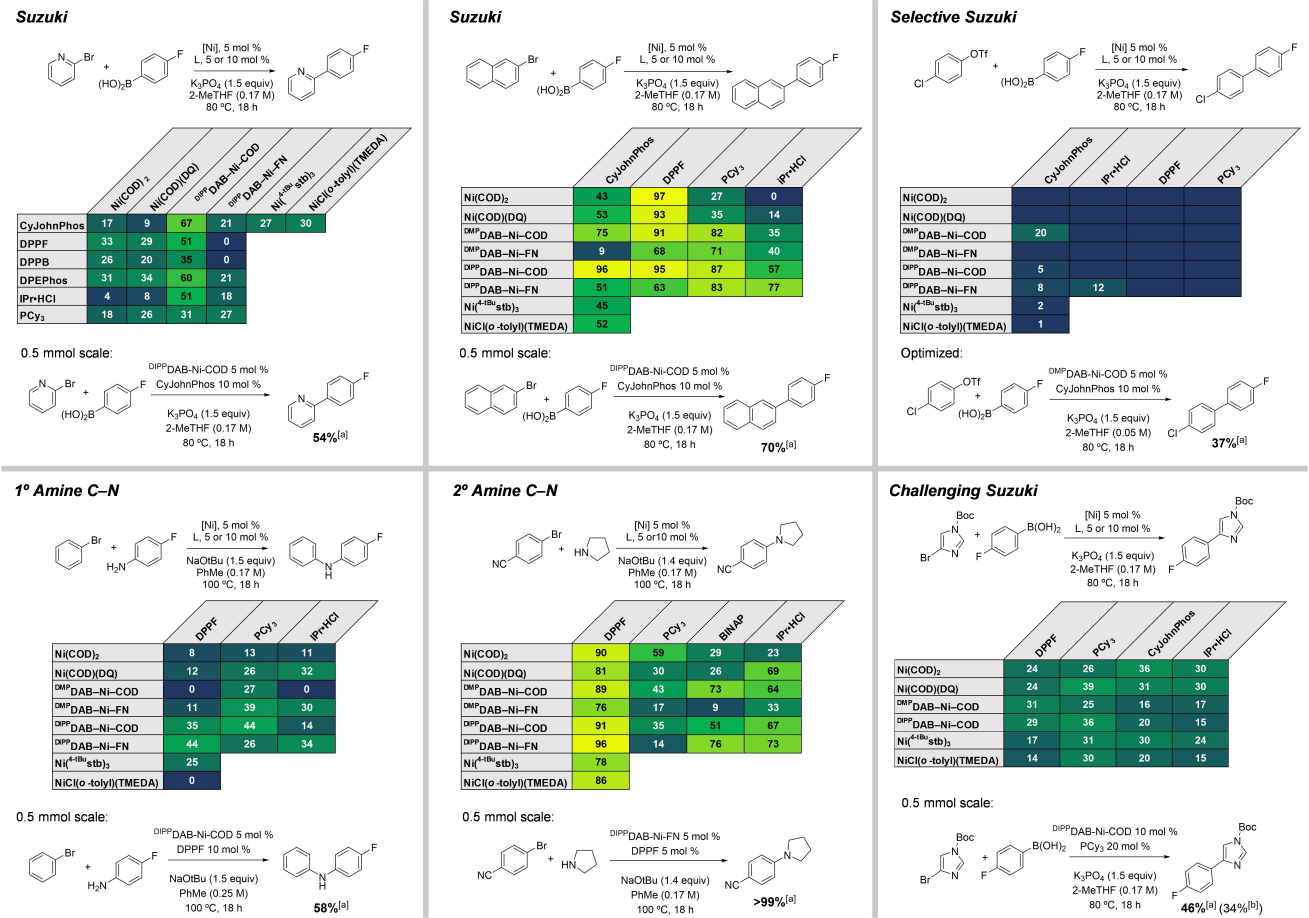
Microscale high‐throughput screening of Ni(0) precatalysts in several Suzuki and C−N coupling reactions. Color gradient indicates solution yield (yellow=100 %, green=50 %, dark blue=0 %); numerical yield values <5 % not shown. Validation reactions performed on a 0.5 mmol scale are displayed below each table. ^[a]^ Solution yield determined by ^19^F or ^1^H NMR spectroscopy with internal standard 4‐benzotrifluoride (for reactions with a fluorine‐containing coupling partner) or 1,3,5‐trimethoxybenzene. ^[b]^ Isolated yield.

With respect to application of **1**–**4** in catalysis, we targeted six coupling reactions for C−C and C−N bond formation using standard phosphine and carbene ligands, and evaluated our precursors against other state‐of‐the‐art Ni sources (Figure 4). First, the Suzuki coupling reaction between 2‐bromopyridine and 4‐fluorobenzeneboronic acid was examined, as the use of nitrogen‐containing heterocycles generally make cross‐couplings more challenging. Ni(COD)_2_ and Ni(COD)(DQ) were compared to **1** and **3** as representative precatalysts across a set of six supporting ligands. Overall, **1** outperformed the other precatalysts in the microscale screening format, with the highest yield observed using CyJohnPhos.^[31]^ To further compare the activity of advanced precursors with the CyJohnPhos supporting ligand, we performed an addition two comparator reactions using Cornella's Ni(^4−tBu^stb)_3_ and the Monfette/Magano/Doyle Ni(II) source NiCl(*o*‐tolyl)(TMEDA). Under these specific conditions, these other two precursors led to lower yields than when using **1**, but are comparable or better than the other Ni sources.Importantly, the identity of the stabilizing ligand clearly has an effect on catalytic activity, with the fumaronitrile‐stabilized **3** underperforming the COD analog **1**. Validation of the **1**/CyJohnPhos hit on 0.5 mmol scale led to the formation of 2‐(4‐fluorophenyl)pyridine in 54 % solution yield without further optimization.

We then examined a broader set of six Ni precursors in a simpler Suzuki coupling between 2‐bromonaphthalene and 4‐fluorobenzeneboronic acid with four supporting ligands. Under these conditions, DPPF performed well with most precursors (91–97 %), though again the fumaronitrile complexes **3** and **4** underperformed (63–68 %).

Notably, DAB‐Ni‐COD complexes **1** and **2** were superior when paired with other ligands – PCy_3_, CyJohnPhos, and the carbene precursor IPr • HCl – with the **1**/CyJohnPhos combination giving 96 % solution yield. We also performed a control experiment using **1** without added ancillary ligand, which gave only 26 % yield. To again compare the activity of the best‐performing precatalyst to other state‐of‐the‐art systems, we tested Ni(^4−tBu^stb)_3_ and NiCl(*o*‐tolyl)(TMEDA) with CyJohnPhos. Under these conditions, both precursors perform similarly to Ni(COD)_2_ and Ni(COD)(DQ) (45–52 %). Validation of the **1**/CyJohnPhos conditions gave the coupling product in 70 % solution yield on 0.5 mmol scale.

Site‐selective cross‐coupling plays a prominent role in synthetic applications and synthesis design, and identification of selective catalysts is important to only facilitate desired transformations with specific (pseudo)halide handles.[^32^, ^33^] We investigated the site‐selective Suzuki coupling of 4‐chlorophenyl triflate and 4‐fluorobenzeneboronic acid under the same conditions as the previous Suzuki coupling HTE plates. Of the two expected products, 4′‐fluoro‐[1,1′‐biphenyl]‐4‐yl trifluoromethane‐sulfonate (from coupling at Cl) and 4‐chloro‐4′‐fluoro‐1,1′‐biphenyl (from coupling at OTf), we only observe selective coupling at OTf to give 4‐chloro‐4′‐fluoro‐1,1′‐biphenyl in appreciable yields in select cases. The data in Figure 4 represents only the solution yields of 4‐chloro‐4′‐fluoro‐1,1′‐biphenyl that are >5 % (see Supporting Information for complete data table). One promising hit was identified in the reaction catalyzed by **2** and CyJohnPhos, providing 4‐chloro‐4′‐fluoro‐1,1′‐biphenyl in a 20 % solution yield. With this ligand, Ni(COD)_2_, Ni(COD)(DQ), Ni(^4−tBu^stb)_3_, or NiCl(*o*‐tolyl)(TMEDA) did not generate either expected product in significant yields. Subsequent validation and optimization of the **2**/CyJohnPhos hit revealed that a lower reaction concentration (0.05 M) gave 37 % solution yield of 4‐chloro‐4′‐fluoro‐1,1′‐biphenyl, with a product ratio of >20 : 1 (no coupling at Cl observed by ^1^H NMR spectroscopy) (Table 2).

**Table 2:** Validation and optimization of the site‐selective Suzuki coupling between 4‐chlorophenyl triflate and 4‐fluorobenzeneboronic acid.^[a]^

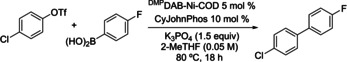		
Entry	Deviation from conditions	NMR Yield(%)^[b]^
1	None	29
2	2 equiv. 4‐fluorobenzeneboronic acid	27
3	3 equiv. 4‐fluorobenzeneboronic acid	16
4	3 equiv. K_3_PO_4_	27
5	T=120 °C	25
6	2.5 % [Ni]/5 % CyJohnPhos	23
7	10 % [Ni]/20 % CyJohnPhos	23
8	12 % CyJohnPhos	19
9	Concentration=0.3 M	19
10	Concentration=0.05 M	37

^[a]^ Conditions: 4‐chlorophenyl triflate (0.05 mmol), 4‐fluorobenzeneboronic acid (1.4 equiv), 2‐MeTHF (0.3 mL) under inert atmosphere. ^[b]^ Solution yields assessed by ^19^F NMR spectroscopy using benzotrifluoride as internal standard.

In a generic primary amine C−N coupling reaction between bromobenzene and 4‐fluoroaniline, we examined a smaller set of three common phosphine ligands. Under these conditions, the highest solution yields were observed with precatalyst/ligand combinations of **1**/PCy_3_ and **3**/DPPF, both at 44 %. Specifically when using DPPF as the ligand under these conditions, Ni(^4−tBu^stb)_3_ generates only 25 % solution yield, and NiCl(*o*‐tolyl)(TMEDA) does not form the product in a measurable amount. Optimization with **1**/DPPF revealed that increasing the ligand loading to 10 %, and increasing the reaction concentration to 0.25 M, leads to 58 % solution yield on 0.5 mmol scale.

A generic secondary amine arylation screen using 4‐bromobenzonitrile and pyrrolidine achieved several high yields with various catalyst systems. DPPF provided >75 % product with all precatalysts under these reaction conditions, with the highest yield observed using **3**/DPPF (96 %). With DPPF, Ni(^4−tBu^stb)_3_ and NiCl(*o*‐tolyl)(TMEDA) are within the range observed for the other Ni(0) sources, indicating this coupling is readily achieved regardless of Ni source. In fact, Cornella and co‐workers recently revealed that such secondary amine C−N couplings can proceed in the absence of ancillary ligand at slightly elevated temperatures.^[34]^

To further probe the reactivity of the DPPF‐based catalyst system, we examined several alternative conditions (Table 3). Using lower catalyst loadings (0.5 mol%) of **3**/DPPF or Ni(COD)_2_/DPPF, we observe 86 % and 52 % yield, respectively, indicating **3** is a more efficient precatalyst for this transformation (Table 3, entries 2 and 3). We also compared **2** as an alternative precatalyst versus Ni(COD)_2_ at higher concentration, with the latter outperforming **2** (entries 4 and 5). However, using the corresponding aryl chlorides revealed that both systems are able to give the product in >99 % solution yield. Subsequent control reactions revealed that in the absence of phosphine ligand, **3** will provide a 45 % yield of product on its own, akin to the previously noted results from Cornella *et al*., who observed that phosphine ligands are not necessary for these C−N couplings.^[34]^ Finally, the reaction will not proceed in the absence of both precatalyst and ligand, ruling out a background S_N_Ar reaction (Table 3, entries 8 and 9).

**Table 3:** Examination of Ni precatalyst reactivity in the secondary amine C−N coupling of pyrrole and 4‐bromobenzonitrile.^[a]^

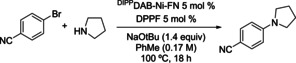		
Entry	Deviation from conditions	NMR Yield(%)^[b]^
1	None	96
2	0.5 mol % **3**, 0.5 mol % DPPF	86
3	0.5 mol % Ni(COD)_2_, 0.5 mol % DPPF	52
4	Ni(COD)_2_ in place of **3**, concentration=0.25 M	92
5	**2** in place of **3**, concentration=0.25 M	76
6	4‐chlorobenzonitrile, Ni(COD)_2_ in place of **3**, concentration=0.25 M	>99
7	4‐chlorobenzonitrile, **2** in place of **3**, concentration=0.25 M	>99
8	No DPPF ligand	45
9	No [Ni], no DPPF ligand	0

^[a]^ Conditions: 4‐bromobenzonitrile (0.06 mmol), pyrrolidine (1.2 equiv), PhMe (0.34 mL) under inert atmosphere. ^[b]^ Solution yields determined by ^1^H NMR spectroscopy with 1,3,5‐trimethoxybenzne as internal standard.

**Figure 5:**
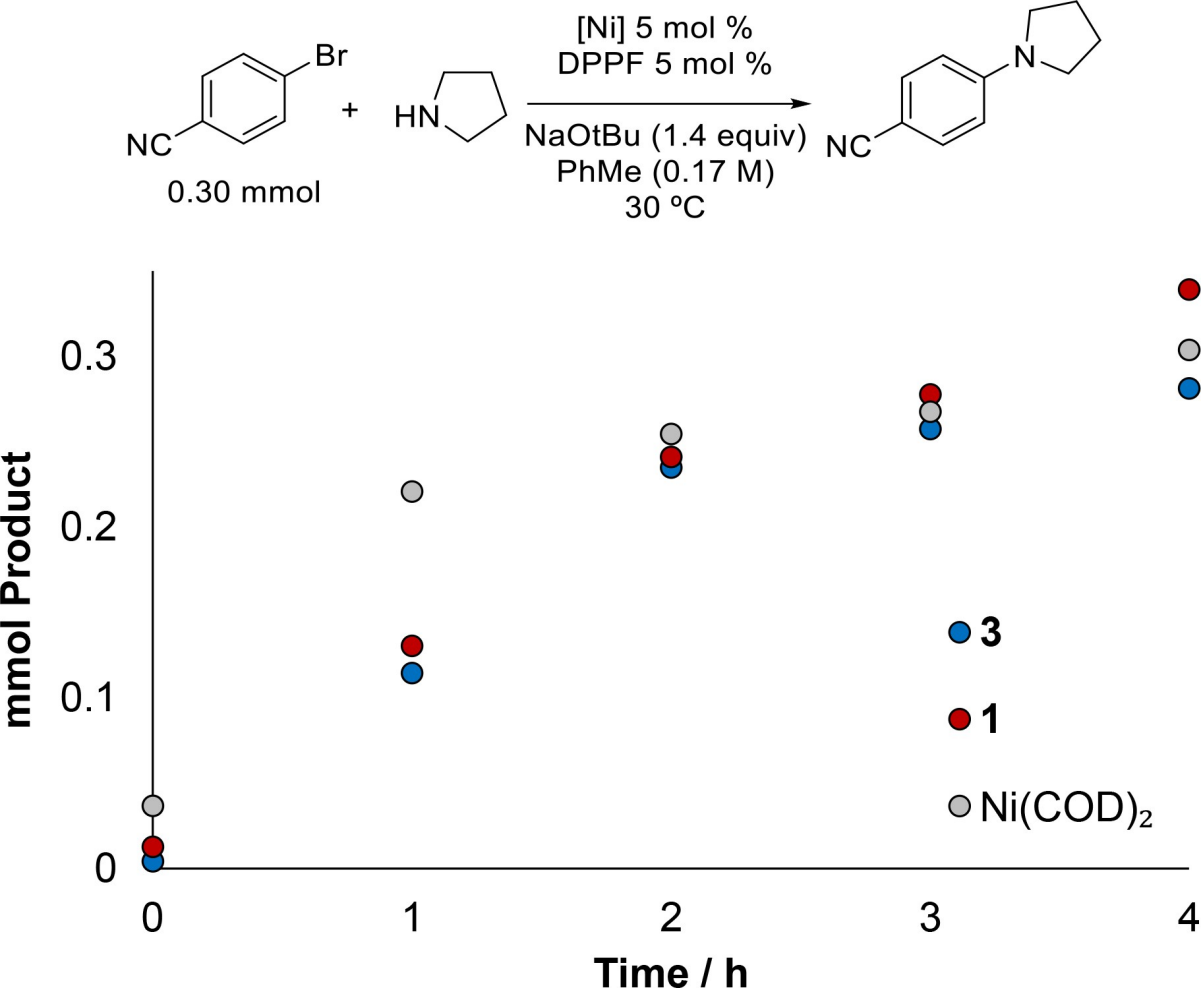
Reaction progress monitoring of the secondary amine C−N coupling between pyrrole and 4‐bromobenzonitrile. Conditions: 4‐bromobenzonitrile (0.3 mmol), pyrrolidine (1.2 equiv), PhMe (1.8 mL) under inert atmosphere at room temperature. Solution yields determined by ^1^H NMR spectroscopy with 1,3,5‐trimethoxybenzene as internal standard.

Taking a closer look into the activation of each precatalyst type, we performed reaction progress monitoring for the pyrrolidine arylation reaction (Figure 5). Ni(COD)_2_, **1** and **3** were used as precatalysts in individual experiments that were monitored over time. Attempts to monitor reaction progress at 100 °C was hampered by the very fast rate of this amination; instead we performed monitoring at 30 °C for 4 h. As per Figure 5, while Ni(COD)_2_ leads to a slightly faster initial rate, all 3 systems reach >94 % product within 4 hours.

Finally, we sought to test the limits of the catalytic reactivity of precatalysts **1** and **2** in a challenging Suzuki coupling involving a five‐membered heterocycle. The Ni‐catalyzed Suzuki coupling of an imidazole substrate has thus far been unreported, and we selected the coupling between 1‐Boc‐4‐bromoimidazole and 4‐fluorobenzeneboronic acid as a test case. The resulting yields on screening scale are similar across the Ni sources, ranging from 14–39 %. The highest yield is observed with Ni(COD)(DQ)/PCy_3_, followed by **1**/PCy_3_ and Ni(COD)_2_/CyJohnPhos, both giving 36 %. Optimization attempts with **1**/PCy_3_ unfortunately did not lead to a significant yield increase (Table 4, entries 2–11). On a 0.5 mmol scale with increased catalyst loading, a 46 % solution yield is achieved, with a 34 % isolated yield after chromatography (entry 13).

**Table 4:** Examination of Ni precatalyst reactivity in a challenging Suzuki coupling of 1‐Boc‐4‐bromoimidazole and 4‐fluorobenzeneboronic acid.^[a]^

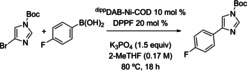		
Entry	Deviation from conditions	NMR Yield(%)^[b]^
1	None	33
2	T=130 °C	0^[c]^
3	Solvent=CPME, T=130 °C	0^[c]^
4	Solvent=PhMe, T=130 °C	0^[c]^
5	Concentration=0.30 M	29
6	Concentration=0.05 M	29
7	[Ni]+L+1‐Boc‐4‐Br‐imidazole pre‐mixed at 80 °C for 1 h	23
8	Slow addition of boronic acid	0
9	Slow addition of 1‐Boc‐4‐Br‐imidazole	22
10	Ar‐BF_3_K instead of Ar‐B(OH)_2_	0
11	Additive: anhydrous ethylene glycol (3 equiv)	0
12	10 % **1**, 20 % DPPF	39
13	10 % **1**, 20 % PCy_3_, 0.50 mmol scale	46 (34)^[d]^

^[a]^ Conditions: 1‐Boc‐4‐Br‐imidazole (0.06 mmol), 4‐fluorobenzeneboronic acid (2.0 equiv), 2‐MeTHF (0.35 mL) under inert atmosphere. ^[b]^ Solution yields determined by ^1^H NMR spectroscopy using 1,3,5‐trimethoxybenzene as internal standard. ^[c]^ 4,4′‐difluoro‐1,1′‐biphenyl was the major product generated. ^[d]^ Isolated yield.

## Conclusions

Overall, we have demonstrated the synthesis and utility of four DAB‐Ni(0) complexes, with further applications in HTE. The solubility and stability profile of each of these precatalysts make them particularly attractive for solution‐dispensing to plate‐based reaction screens, a distinct advantage over other commonly used Ni(0) sources. Of the four DAB‐Ni(0) complexes explored, ^DIPP^DAB‐Ni‐COD (**1**) emerged as a top contender in both Suzuki and C−N coupling reactions, however ^DMP^DAB‐Ni‐COD (**2**) demonstrated greater chemoselectivity. Not only is the synthesis of **1**–**4** easily scalable, but their catalytic activity is maintained in larger scale reactions as well. While these initial results show the promise of these precatalysts in Suzuki and C−N coupling, further investigations are necessary to fully explore the scope and generality of these systems in complex molecule synthesis. These studies are currently ongoing in our laboratories.

## Experimental

The following procedures are representative syntheses of **1** and **3**. Further details and characterization data are provided in the Supporting Information.

### Synthesis of ^DIPP^DAB‐Ni‐COD (1)

*From Ni(COD)*_*2*_ : Outside the glovebox, an oven‐dried 8‐dram vial with a Teflon‐lined cap equipped with a stir bar was charged with diimine ligand (^DIPP^DAB: 846.1 mg, 2.2 mmol). The vial was brought into the glovebox, and then charged with Ni(COD)_2_ (600 mg, 2.2 mmol). Anhydrous toluene was then added using an oven‐dried graduated cylinder (0.14 M reaction concentration; 16 mL). The vial was capped and stirred for 18 h inside the glovebox. The dark brown/black solution was then filtered through Celite, which was rinsed through with excess anhydrous toluene, and subsequently dried *in vacuo* inside the glovebox to obtain the desired complex **1** (92 % yield, 1.10 g) as a dark brown/black solid, which based on NMR spectroscopy did not require further purification.

*From (* ^*DIPP*^ *DAB)NiBr*_*2*_ : Inside the glovebox, an oven‐dried 8‐dram vial equipped with a stir bar was charged with (^DIPP^DAB)NiBr_2_ ^[35]^ (415 mg, 0.7 mmol) and small pieces of Na metal (165 mg, 7.2 mmol). The vial was sealed with a septum cap (pressure relief cap). A separate 4‐dram vial was charged with anhydrous THF (6.5 mL) and 1,5‐cyclooctadiene (380 mg, 430 μL, 3.5 mmol). Both vials were removed from the glovebox. The 8‐dram vial containing (^DIPP^DAB)NiBr_2_ and Na was placed into an ice bath at 0 °C. A 27.5G needle/syringe was used to add the entire solution of THF/COD through the septum while stirring at 0 °C. After 1 h the vial was removed from the ice bath and allowed to stir at room temperature. After 45 mins, the solution turned violet and the (^DIPP^DAB)NiBr_2_ had entirely dissolved. After 2 hours the solution turned dark red/brown with no evidence of the violet colour. At this point, the vial was brought back into the glovebox. The suspension was filtered through a small bed of Celite, which was then rinsed with excess anhydrous toluene. The filtrate was then dried *in vacuo* to obtain **1** as a dark brown/black solid in 94 % isolated yield (0.355 g), which based on NMR spectroscopy did not require further purification.

### Synthesis of ^DIPP^DAB‐Ni‐FN (3)

Inside the glovebox, ^DIPP^DAB‐Ni‐COD (**1**) (150 mg, 0.28 mmol) was weighed into an oven‐dried 4‐dram vial equipped with a stir bar. Fumaronitrile was then added (22 mg, 0.28 mmol). A 100–1000 mL micropipette was then used to add anhydrous toluene (0.1 M; 2.7 mL). The vial was capped with a Teflon‐lined screw cap, and the mixture stirred for 18 h inside the glovebox. The dark red/black solution was then dried *in vacuo* inside the glovebox to remove solvent and displaced COD, giving the desired complex **3** in 98 % yield (141 mg), which based on NMR spectroscopy did not require further purification.

## Supporting Information

The authors have cited additional references within the Supporting Information (Ref. [35–42]).

## Conflict of Interests

The authors declare no conflict of interest.

1

## Supporting information

As a service to our authors and readers, this journal provides supporting information supplied by the authors. Such materials are peer reviewed and may be re‐organized for online delivery, but are not copy‐edited or typeset. Technical support issues arising from supporting information (other than missing files) should be addressed to the authors.

Supporting Information

## Data Availability

The data that support the findings of this study are available in the supplementary material of this article.
