# Supplementary material for: Easily Accessible and Solution‐Stable Ni(0) Precatalysts for High‐Throughput Experimentation
Source: Chemistry. 2025 Jan 28;31(12):e202403960. doi: 10.1002/chem.202403960 (PMC11855265; doi:10.1002/chem.202403960)
Supplement: Supplementary file 1 — Supporting Information [file CHEM-31-e202403960-s001.pdf]

Chemistry–A European Journal

Supporting Information

# Easily Accessible and Solution-Stable Ni(0) Precatalysts for High-Throughput Experimentation

Gilian T. Thomas, Odhran D. Cruise, Daelin Peel-Smith, Nahiane Pipaón Fernández, Charles Killeen, and David C. Leitch*

# Supporting Information

# Easily Accessible and Solution-Stable Ni(0) Precatalysts for High-Throughput Experimentation

Gilian T. Thomas,[a] Odhran D. Cruise,[a] Daelin Peel-Smith,[a] Nahiane Pipaón Fernández,[a] Charles Killeen,[a] and David C. Leitch[a]*

[a]Department of Chemistry, University of Victoria, Victoria, British Columbia V8P 5C2, Canada.

*dcleitch@uvic.ca

## Table of Contents

**I. GENERAL CONSIDERATIONS ........ 2**

Materials ........ 2
Techniques ........ 2
Analysis and Spectroscopy ........ 2

**II. SYNTHESIS AND CHARACTERIZATION OF NICKEL COMPLEXES ........ 3**

Synthesis of $^{DIPP}$DAB–Ni–COD (1) and $^{DMP}$DAB–Ni–COD (2) ........ 3
Synthesis of $^{DIPP}$DAB–Ni–FN (3) and $^{DMP}$DAB–Ni–FN (4) ........ 4

**III. HIGH-THROUGHPUT EXPERIMENTATION ........ 6**

**IV. VALIDATION SYNTHESIS PROCEDURES ........ 10**

**V. SOLUBILITY AND STABILITY OF NICKEL COMPLEXES ........ 12**

$^{DIPP}$DAB–Ni–COD ........ 13
$^{DMP}$DAB–Ni–COD ........ 16
$^{DIPP}$DAB–Ni–FN ........ 19
$^{DMP}$DAB–Ni–FN ........ 22

**VI. LIGAND EXCHANGE REACTION MONITORING EXPERIMENT ........ 25**

**VII. SPECTRA ........ 27**

**VIII. REFERENCES ........ 41**

# I. General Considerations

## Materials

All solvents and common organic reagents were purchased from commercial suppliers and used without further purification. All solvents used in these procedures were purchased as anhydrous from MilliporeSigma and used as received, stored under inert atmosphere in a nitrogen-filled MBraun glovebox. $Ni(COD)_2$ was purchased from Strem Chemicals and used as received. $(DME)NiBr_2$ was purchased from MilliporeSigma and used as received. $(^{DIPP}DAB)NiBr_2$ was synthesized according to the literature.[1] *N,N'*-bis(2,6-dimethylphenyl)ethan-1,2-diimine ($^{DMP}$DAB) and *N,N'*- bis(2,6-diisopropyl)ethan-1,2-diimine ($^{DIPP}$DAB) were prepared using a reported procedure.[2] All phosphine ligands were purchased from Strem Chemicals and used as received. *tert*-Butyl 4-bromo-1*H*-imidazole-1-carboxylate and 4-chlorophenyl trifluoromethanesulfonate were synthesized according to literature procedures.[3,4]

## Techniques

All air-free manipulations were performed under a dry nitrogen atmosphere using an MBraun glovebox. High-throughput experimentation was performed using 1 mL capacity glass shell vials in sealable aluminum reaction blocks purchased from Analytical Sales. Heating/stirring was achieved using rare-earth magnetic tumble stirrers acquired from V&P Scientific. Centrifugal evaporation was performed using a Genevac EZ-2 (HCl compatible).

Automated chromatography purification was performed using Biotage® Selekt SEL-2SW instruments using Sfär Silica (60 µm) columns.

## Analysis and Spectroscopy

All NMR spectra were acquired on either a Bruker AVANCE 300 MHz spectrometer or a Bruker AVANCE Neo 500 MHz spectrometer. All $^{1}$H and $^{13}$C NMR chemical shifts are calibrated to residual protio-solvents. All NMR spectroscopic data is processed using Bruker TopSpin 3.6.1.

High-resolution electrospray ionization mass spectrometric analysis was performed using a Thermo Scientific Ultimate 3000 ESI-Orbitrap Exactive Plus, or a Waters Synapt G2-Si.

Elemental analysis was performed at the CENTC Elemental Analysis Facility at the University of Rochester. Microanalysis samples were weighed with a PerkinElmer Model AD6000 Autobalance and their compositions were determined with a PerkinElmer 2400 Series II Analyzer. All samples were handled under argon in a VAC Atmospheres glovebox.

## II. Synthesis and Characterization of Nickel Complexes

### Synthesis of $^{DIPP}$DAB–Ni–COD (1) and $^{DMP}$DAB–Ni–COD (2)

*From Ni(COD)$_2$*: Outside the glovebox, an oven-dried 8-dram vial with a Teflon-lined cap equipped with a stir bar was charged with diimine ligand ($^{DIPP}$DAB: 846.1 mg, 2.2 mmol; or $^{DMP}$DAB: 197.3 mg, 0.75 mmol). The vial was brought into the glovebox, and Ni(COD)$_2$ was weighed into the vial (for $^{DIPP}$DAB–Ni–COD: 600 mg, 2.2 mmol; for $^{DMP}$DAB–Ni–COD: 199.3 mg, 0.7 mmol). Anhydrous toluene was then added using an oven-dried graduated cylinder (0.14 M reaction concentration; for $^{DIPP}$DAB–Ni–COD: 16 mL; for $^{DMP}$DAB–Ni–COD: 5 mL). The vial was capped and stirred for 18 h inside the glovebox. The dark brown/black solution was then filtered through Celite, which was rinsed through with excess anhydrous toluene, and subsequently dried *in vacuo* inside the glovebox to obtain the desired complex (**1**: 92% yield, 1.10 g; **2**: 97% yield, 0.307 g ) as a dark brown/black solid without further purification. NMR analysis samples were always filtered through a small Celite plug to ensure any trace paramagnetic impurities were removed prior to analysis.

*From ($^{DIPP}$DAB)NiBr$_2$*: Inside the glovebox, an oven-dried 8-dram vial equipped with a stir bar was charged with ($^{DIPP}$DAB)NiBr$_2$[1] (415 mg, 0.7 mmol) and small pieces of Na (165 mg, 7.2 mmol). The vial was sealed with a septum cap (pressure relief cap). A separate 4-dram vial was charged with anhydrous THF (6.5 mL) and 430 μL of 1,5-cyclooctadiene. Both vials were removed from the glovebox. The 8-dram vial containing ($^{DIPP}$DAB)NiBr$_2$ and Na was put into an ice bath at 0 ºC. A 27.5G needle/syringe was used to add the entire solution of THF/COD through the septum while stirring at 0 ºC. After 1 h the vial was removed from the ice bath and allowed to stir at room temperature. After 45 mins, the solution turned violet and the ($^{DIPP}$DAB)NiBr$_2$ had entirely dissolved. After 2 hours the solution turned dark red/brown with no evidence of the violet colour. At this point, the vial was brought back into the glovebox and was then filtered through a small bed of Celite and rinsed through with excess anhydrous toluene. The filtrate was then dried *in vacuo* to obtain **1** as a dark brown/black solid in 94% isolated yield (0.355 g). NMR analysis samples were always filtered through a small Celite plug to ensure any trace paramagnetic impurities were removed prior to analysis.

**Note**: the specific surface area / surface chemistry of the sodium used may lead to changes in the time to reach complete reduction. The colour change from violet to red/brown is a reliable indicator of the reaction endpoint. If the reaction mixture is exposed to Na for too long, decomposition of **1** will occur.

$^{DIPP}$DAB–Ni–COD, **1**[5]

$^{1}$H NMR (500 MHz; $C_6D_6$) δ 7.90 (s, 1H), 7.31-7.23 (m, 6H), 3.91 (s, 4H), 3.36-3.30 (m, 4H), 2.42-2.39 (m, 4H), 1.41 (d, 4H), 1.38 (d, 12H), 1.10 (d, 12H).

$^{13}$C NMR (125 MHz; $C_6D_6$) δ 153.8, 141.2, 139.4, 125.2, 122.8, 88.5, 30.3, 27.6, 26.2, 22.7.

IR (ATR, solid): 3061, 2957, 2666, 1625, 1460 $cm^{-1}$.

HR-MS (ESI): theoretical ($C_{34}H_{48}N_2Ni$) $[M]^+$ *m/z* 542.3171; found *m/z* 542.3195.

Elemental analysis (CHN): theoretical ($C_{34}H_{48}N_2Ni$): 75.14, 8.90, 5.15; found: 75.10, 9.08, 5.21.

$^{DMP}$DAB–Ni–COD, **2**

$^{1}$H NMR (300 MHz; $C_6D_6$) δ 7.69 (s, 2H), 7.13 (s, 6H), 3.72 (s, 4H), 2.36 (m, 4H), 2.18 (s, 12H), 1.42-1.37 (m, 4H).

$^{13}$C NMR (125 MHz; $C_6D_6$) δ 156.4, 140.4, 138.0, 124.1, 87.7, 30.3, 19.5, 17.8.

IR (ATR, solid): 3001, 2950, 2917, 1617, 1487 $cm^{-1}$.

HR-MS (ESI) theoretical ($C_{26}H_{32}N_2Ni$) $[M]^+$ *m/z* 430.1919; found *m/z* 430.1983.

Elemental analysis (CHN): theoretical ($C_{26}H_{32}N_2Ni$): 72.42, 7.48, 6.50; found: 71.66, 7.49, 6.49.

## Synthesis of $^{DIPP}$DAB–Ni–FN (3) and $^{DMP}$DAB–Ni–FN (4)

Inside the glovebox, $^{R}$DAB–Ni–COD ($^{DIPP}$DAB–Ni–COD: 150 mg, 0.28 mmol; $^{DMP}$DAB–Ni–COD: 150 mg, 0.35 mmol) was weighed into an oven-dried 4-dram vial equipped with a stir bar. Fumaronitrile was then weighed into the vial as well (for $^{DIPP}$DAB–Ni–FN: 22 mg, 0.28 mmol; for $^{DMP}$DAB–Ni–FN: 27 mg, 0.35 mmol). A 100-1000 mL micropipette was then used to add anhydrous toluene (0.1 M; for $^{DIPP}$DAB–Ni–FN: 2.7 mL; for $^{DMP}$DAB–Ni–FN: 3.4 mL). The vial was capped and stirred for 18 h inside the glovebox. The dark red/black solution was then dried *in vacuo* inside the glovebox to obtain the desired complex (**3**: 98%, 141 mg; **4**: 93%, 130 mg) without further purification. NMR samples were always filtered through Celite to ensure any paramagnetic material was removed prior to analysis.

$^{DIPP}$DAB–Ni–FN, **3**

$^{1}$H NMR (300 MHz; $C_6D_6$) δ 8.17 (s, 2H), 7.12-7.09 (m, 6H), 3.12 (q, 4H), 1.17 (s, 12H), 1.15 (s, 12H), 1.04-1.02 (m, 2H).

$^{13}$C NMR (125 MHz; $C_6D_6$) δ 163.11, 148.57, 136.54, 125.23, 123.24, 28.15, 23.10.

IR (ATR, solid): 3063, 2961, 2924, 2667, 2220, 2195, 1625, 1587 $cm^{-1}$.

Elemental analysis (CHN): theoretical ($C_{30}H_{38}N_4Ni$): 70.19, 7.46, 10.91 (calc'd); 70.10, 7.82, 10.22.

$^{DMP}$DAB–Ni–FN, **4**

$^{1}$H NMR (300 MHz; $C_6D_6$) δ 7.94 (s, 2H), 7.06 (t, 2H), 6.83 (d, 4H), 2.24 (s, 12H), 2.20 (br s, 2H).

$^{13}$C NMR (125 MHz; $C_6D_6$) δ 158.4, 138.0, 129.1, 128.2, 124.0, 19.5.

IR (ATR, solid): 3064, 2918, 2219, 2196 1625, 1587 $cm^{-1}$.

Repeated attempts to acquire elemental analysis (CHN) for **4** ($C_{22}H_{22}N_4Ni$) resulted in consistently low %C.

## III. High-Throughput Experimentation

General Screening Procedure: High-throughput experimentation was performed using 1 mL capacity glass shell vials in sealable aluminum reaction blocks purchased from Analytical Sales. In a nitrogen-filled glovebox, stock solutions of soluble reaction components were prepared. Insoluble reagents were added as solids to respective vials. Stock solutions were then divided into respective vials using a 20-200 μL micropipette such that the aryl halide content (limiting reagent) was 0.05 mmol. Finally, appropriate solvent was added to every vial to reach the desired final concentration. The plate was sealed, and the mixtures heated/agitated using a tumble stirrer at the selected temperature for 18 h. The plate was then removed from the stirrer, the mixtures were filtered through Celite, and the solvent was then removed under reduced pressure using centrifugal evaporation. NMR spectroscopic analysis was then performed in $CDCl_3$.

The coupling of 2-bromopyridine and (4-fluorophenyl)boronic acid (Suzuki Coupling in Figure 3) was set up following this procedure:

In a nitrogen-filled glovebox, stock solutions of substrates 2-bromopyridine (1 M, 0.284 g in 1.8 mL 2-MeTHF) and (4-fluorophenyl)boronic acid (1.2 M, 0.302 g in 1.8 mL 2-MeTHF) were prepared. Stock solutions of $Ni(COD)_2$, $^{DIPP}$DAB–Ni–COD and $^{DIPP}$DAB–Ni–FN, were prepared (0.05 M in PhMe), and stock solutions of DPPF (0.05 M), DPPB (0.05 M), DPEPhos (0.05 M), $PCy_3$ (0.1 M), and CyJohnPhos (0.1 M) were also prepared in 2-MeTHF. Ni(COD)(DQ) and IPr•HCl were weighed and added as solids to respective vials. Ligand-containing stock solutions were added to respective vials (50 μL each), followed by Ni-containing stock solutions (50 μL each). $K_3PO_4$ (15.9 mg, 1.5 equiv) was then added to each vial using a plastic calibrated scoop (TWD TradeWinds, ASPS-01, Disposable Antistatic Polypropylene Sample Transfer Scoop, 1-3 mg capacity). Stock solutions of substrates were then added to each vial (50 μL each), followed by a further portion of 2-MeTHF to reach a total volume of 300 μL (0.17 M). The plate was sealed, and the mixtures heated/agitated using a tumble stirrer at 100 ºC for 18 h. The plate was then removed from the stirrer, each mixture was filtered through a Celite plug, and the solvent was then removed under reduced pressure using centrifugal evaporation. NMR spectroscopic analysis was then performed in $CDCl_3$.

The coupling of 2-bromonaphthalene and (4-fluorophenyl)boronic acid (Suzuki Coupling in Figure 3) was set up following this procedure:

In a nitrogen-filled glovebox, stock solutions of substrates 2-bromonaphthalene (1 M, 0.374 g in 1.8 mL 2-MeTHF) and (4-fluorophenyl)boronic acid (1.2 M, 0.302 g in 1.8 mL 2-MeTHF) were prepared. Stock solutions of $Ni(COD)_2$, $^{DIPP}$DAB–Ni–COD, $^{DMP}$DAB–Ni–COD, $^{DIPP}$DAB–Ni–FN, and $^{DMP}$DAB–Ni–FN, were prepared (0.05 M in 2-MeTHF), and stock solutions of DPPF (0.05 M), $PCy_3$ (0.1 M) and CyJohnPhos (0.1 M) were also prepared in 2-MeTHF. Ni(COD)(DQ) and IPr•HCl were weighed and added as solids to respective vials. Ligand-containing stock solutions were added to respective vials (50 μL each), followed by Ni-containing stock solutions (50 μL each). $K_3PO_4$ (15.9 mg, 1.5 equiv) was then added to each vial using a plastic calibrated scoop (TWD TradeWinds, ASPS-

01, Disposable Antistatic Polypropylene Sample Transfer Scoop, 1-3 mg capacity). Stock solutions of substrates were then added to each vial (50 μL each), followed by a further portion of 2-MeTHF to reach a total volume of 300 μL (0.17 M). The plate was sealed, and the mixtures heated/agitated using a tumble stirrer at 100 ºC for 18 h. The plate was then removed from the stirrer, each mixture was filtered through a Celite plug, and the solvent was then removed under reduced pressure using centrifugal evaporation. NMR spectroscopic analysis was then performed in $CDCl_3$.

The coupling of 4-chlorophenyl trifluoromethanesulfonate and (4-fluorophenyl)boronic acid (Selective Suzuki Coupling in Figure 3) was set up following this procedure:

In a nitrogen-filled glovebox, stock solutions of substrates 4-chlorophenyl trifluoromethanesulfonate (1 M, 0.469 g in 1.8 mL 2-MeTHF) and (4-fluorophenyl)boronic acid (1.2 M, 0.302 g in 1.8 mL 2-MeTHF) were prepared. Stock solutions of $Ni(COD)_2$, $^{DIPP}$DAB–Ni–COD, $^{DMP}$DAB–Ni–COD, $^{DIPP}$DAB–Ni–FN, and $^{DMP}$DAB–Ni–FN, were prepared (0.05 M in 2-MeTHF), and stock solutions of DPPF (0.05 M), $PCy_3$ (0.1 M) and CyJohnPhos (0.1 M) were also prepared in 2-MeTHF. Ni(COD)(DQ) and IPr•HCl were weighed and added as solids to respective vials. Ligand-containing stock solutions were added to respective vials (50 μL each), followed by Ni-containing stock solutions (50 μL each). $K_3PO_4$ (15.9 mg, 1.5 equiv) was then added to each vial using a plastic calibrated scoop (TWD TradeWinds, ASPS-01, Disposable Antistatic Polypropylene Sample Transfer Scoop, 1-3 mg capacity). Stock solutions of substrates were then added to each vial (50 μL each), followed by a further portion of 2-MeTHF to reach a total volume of 300 μL (0.17 M). The plate was sealed, and the mixtures heated/agitated using a tumble stirrer at 100 ºC for 18 h. The plate was then removed from the stirrer, each mixture was filtered through a Celite plug, and the solvent was then removed under reduced pressure using centrifugal evaporation. NMR spectroscopic analysis was then performed in $CDCl_3$.

The coupling of bromobenzene and 4-fluoroaniline (1º Amine C–N Coupling in Figure 3) was set up following this procedure:

In a nitrogen-filled glovebox, stock solutions of substrates bromobenzene (1 M, 0.283 g in 1.8 mL PhMe) and 4-fluoroaniline (1.2 M, 0.240 g in 1.8 mL PhMe) were prepared. Stock solutions of $Ni(COD)_2$, $^{DIPP}$DAB–Ni–COD, $^{DMP}$DAB–Ni–COD, $^{DIPP}$DAB–Ni–FN, and $^{DMP}$DAB–Ni–FN, were prepared (0.05 M in PhMe), and stock solutions of DPPF (0.05 M) and $PCy_3$ (0.1 M) were also prepared in PhMe. Ni(COD)(DQ) and IPr•HCl were weighed and added as solids to respective vials. Ligand-containing stock solutions were added to respective vials (50 μL each), followed by Ni-containing stock solutions (50 μL each). NaO*t*Bu (7.2 mg, 1.5 equiv) was then added to each vial using a plastic calibrated scoop (TWD TradeWinds, ASPS-01, Disposable Antistatic Polypropylene Sample Transfer Scoop, 1-3 mg capacity). Stock solutions of substrates were then added to each vial (50 μL each), followed by a further portion of PhMe to reach a total volume of 300 μL (0.17 M). The plate was sealed, and the mixtures heated/agitated using a tumble stirrer at 100 ºC for 18 h. The plate was then removed from the stirrer, each mixture was filtered through a Celite plug, and the solvent was then removed under reduced pressure using centrifugal evaporation. NMR spectroscopic analysis was then performed in $CDCl_3$.

The coupling of 4-bromobenzonitrile and pyrrolidine (2º amine C–N coupling screen in Figure 3) was set up following this procedure:

In a nitrogen-filled glovebox, stock solutions of substrates 4-bromobenzonitrile (1 M, 0.328 g in 1.8 mL PhMe) and pyrrolidine (1.2 M, 0.154 g in 1.8 mL PhMe) were prepared. Stock solutions of $Ni(COD)_2$, $^{DIPP}$DAB–Ni–COD, $^{DMP}$DAB–Ni–COD, $^{DIPP}$DAB–Ni–FN, and $^{DMP}$DAB–Ni–FN were prepared (0.05 M in PhMe), and stock solutions of $PCy_3$ (0.1 M), DPPF (0.05 M), and BINAP (0.05 M) were also prepared in PhMe. Ni(COD)(DQ) and IPr•HCl were weighed and added as solids to respective vials. Ligand-containing stock solutions were added to respective vials (50 μL each), followed by Ni-containing stock solutions (50 μL each). NaOtBu (6.7 mg, 1.4 equiv) was then added to each vial using a plastic calibrated scoop (TWD TradeWinds, ASPS-01, Disposable Antistatic Polypropylene Sample Transfer Scoop, 1-3 mg capacity). Stock solutions of substrates were then added to each vial (50 μL each), followed by a further portion of PhMe to reach a total volume of 300 μL (0.17 M). The plate was sealed, and the mixtures heated/agitated using a tumble stirrer at 100 ºC for 18 h. The plate was then removed from the stirrer, each mixture was filtered through a Celite plug, and the solvent was then removed under reduced pressure using centrifugal evaporation. NMR spectroscopic analysis was then performed in $CDCl_3$.

The coupling of *tert*-butyl 4-bromo-1*H*-imidazole-1-carboxylate and (4-fluorophenyl)boronic acid (Challenging Suzuki Coupling in Figure 3) was set up following this procedure:

In a nitrogen-filled glovebox, stock solutions of substrates *tert*-butyl 4-bromo-1*H*-imidazole-1-carboxylate (1 M, 0.297 g in 1.8 mL 2-MeTHF) and (4-fluorophenyl)boronic acid (1.2 M, 0.302 g in 1.8 mL 2-MeTHF) were prepared. Stock solutions of $Ni(COD)_2$, $Ni(^{4\text{-tBu}}stb)_3$, NiCl(*o*-tolyl)(TMEDA), $^{DIPP}$DAB–Ni–COD and $^{DMP}$DAB–Ni–COD were prepared (0.05 M in 2-MeTHF), and stock solutions of DPPF (0.05 M), $PCy_3$ (0.1 M) and CyJohnPhos (0.1 M) were also prepared in 2-MeTHF. Ni(COD)(DQ) and IPr•HCl were weighed and added as solids to respective vials. Ligand-containing stock solutions were added to respective vials (50 μL each), followed by Ni-containing stock solutions (50 μL each). $K_3PO_4$ (15.9 mg, 1.5 equiv) was then added to each vial using a plastic calibrated scoop (TWD TradeWinds, ASPS-01, Disposable Antistatic Polypropylene Sample Transfer Scoop, 1-3 mg capacity). Stock solutions of substrates were then added to each vial (50 μL each), followed by a further portion of 2-MeTHF to reach a total volume of 300 μL (0.17 M). The plate was sealed, and the mixtures heated/agitated using a tumble stirrer at 100 ºC for 18 h. The plate was then removed from the stirrer, each mixture was filtered through a Celite plug, and the solvent was then removed under reduced pressure using centrifugal evaporation. NMR spectroscopic analysis was then performed in $CDCl_3$.

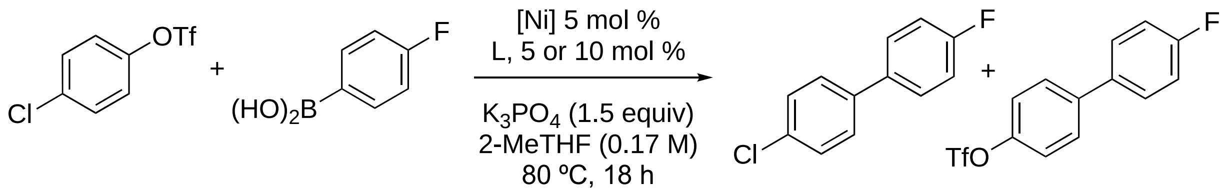


| | DPPF | $PCy_3$ | CyJohnPhos | Ipr HCl |
|---|---|---|---|---|
| **$Ni(COD)_2$** | 2 / 0.3 | 2 / 0.3 | 2 / 0.1 | 0.4 / 0 |
| **Ni(COD)(DQ)** | 1 / 0.2 | 2 / 0.4 | 1 / 0.1 | 0.5 / 0.1 |
| **$^{dmp}$DAB-Ni-COD** | 4 / 0.5 | 3 / 0.4 | 20 / 0.6 | 2.5 / 3 |
| **$^{dmp}$DAB-Ni-FN** | 1 / 0 | 0.1 / 0 | 1 / 0 | 2 / 0 |
| **$^{dipp}$DAB-Ni-COD** | 2 / 0.3 | 2 / 0.4 | 5 / 0.4 | 1 / 0.1 |
| **$^{dipp}$DAB-Ni-FN** | 2 / 0.2 | 1.5 / 0.2 | 8 / 0.2 | 12 / 0.5 |

**Figure S1.** Detailed high-throughput experimentation results for the selectivity screen of the reaction between 4-chlorophenyl trifluoromethanesulfonate and 4-fluorobenzene boronic acid. These are displayed as solution yields by $^{19}F$ NMR spectroscopy compared to benzotrifluoride internal standard, and represented as: %4-F-4'-Cl-biphenyl product / %4-F-4'-OTf-biphenyl product.

# IV. Validation Synthesis Procedures

2-(4-fluorophenyl)pyridine: Outside the glovebox, 4-fluorobenzeneboronic acid (136 mg, 0.97 mmol) and powdered $K_3PO_4$ (154 mg, 0.7 mmol) were weighed into an oven-dried 4-dram vial. The vial was brought into the glovebox, and $^{DIPP}$DAB–Ni–COD (13 mg, 0.02 mmol) and CyJohnPhos (17 mg, 0.05 mmol) were weighed into the vial. Anhydrous 2-MeTHF (3 mL) was added using a 1000 μL micropipette, followed by 2-bromopyridine (77 mg, 0.5 mmol). The vial was sealed with a Teflon-lined cap, and removed from the glovebox. The vial was placed on a tumble stirrer at 80 ºC for 18 h, after which time the reaction mixture was filtered through a small Celite plug, and rinsed through with ethyl acetate. The solvent was then removed under reduced pressure using centrifugal evaporation, and a $^1$H NMR spectrum was taken in $CDCl_3$. The solution yield was calculated relative to a 1,3,5-trimethoxybenzene internal standard as 54%. NMR spectroscopic characteristics are identical to those reported.[6]

2-(4-fluorophenyl)naphthalene: Outside the glovebox, 4-fluorobenzeneboronic acid (141 mg, 1.0 mmol), 4-bromonaphthalene (104 mg, 0.5 mmol) and powdered $K_3PO_4$ (161 mg, 0.76 mmol) were weighed into an oven-dried 4-dram vial. The vial was brought into the glovebox, and $^{DIPP}$DAB–Ni–COD (14 mg, 0.03 mmol) and CyJohnPhos (18 mg, 0.05 mmol) were weighed into the vial. Anhydrous 2-MeTHF (3 mL) was added using a 1000 μL micropipette. The vial was sealed with a Teflon-lined cap, and removed from the glovebox. The vial was placed on a tumble stirrer at 80 ºC for 18 h, after which time the reaction mixture was filtered through a small Celite plug, and rinsed through with ethyl acetate. The solvent was then removed under reduced pressure using centrifugal evaporation, and a $^1$H NMR spectrum was taken in $CDCl_3$. The solution yield was calculated relative to a 1,3,5-trimethoxybenzene internal standard as 70%. NMR spectroscopic characteristics are identical to those reported.[7]

4-fluoro-*N*-phenylaniline: Inside the glovebox $^{DIPP}$DAB–Ni–COD (13 mg, 0.02 mmol), DPPF (26 mg, 0.05 mmol), and sodium *tert*-butoxide (68 mg, 0.7 mmol) were weighed into an oven-dried 4-dram vial. Anhydrous toluene (2 mL) was added using a 1000 μL micropipette, followed by bromobenzene (74 mg, 0.5 mmol) and 4-fluoroaniline (63 mg, 0.6 mmol). The vial was sealed with a Teflon-lined cap, and removed from the glovebox. The vial was placed on a tumble stirrer at 100 ºC for 18 h, after which time the reaction mixture was filtered through a small Celite plug, and rinsed through with ethyl acetate. The solvent was then removed under reduced pressure using centrifugal evaporation, and a $^{19}$F NMR spectrum was taken in $CDCl_3$. The solution yield was calculated relative to a 4-benzotrifluoride internal standard as 58%. NMR spectroscopic characteristics are identical to those reported.[8]

4-(pyrrolidin-1-yl)benzonitrile: Outside the glovebox, 4-bromobenzonitrile (91 mg, 0.5 mmol) was weighed into an oven-dried 4-dram vial. The vial was brought into the glovebox, and $^{DIPP}$DAB–Ni–COD (13 mg, 0.03 mmol), DPPF (14 mg, 0.03 mmol), and sodium *tert*-butoxide (68 mg, 0.7 mmol) were weighed into the vial. Anhydrous toluene (3 mL) was added using a 1000 μL micropipette, followed by pyrrolidine (43 mg, 0.6 mmol). The vial was sealed with a Teflon-lined cap, and removed from the glovebox. The vial was placed on a tumble stirrer at 100 ºC for 18 h, after which time the reaction mixture

was filtered through a small Celite plug, and rinsed through with ethyl acetate. The solvent was then removed under reduced pressure using centrifugal evaporation, and a $^{1}H$ NMR spectrum was taken in $CDCl_3$. The solution yield was calculated relative to a 1,3,5-trimethoxybenzene internal standard as 108%.[9]

*tert*-Butyl 4-(4-fluorophenyl)-1*H*-imidazole-1-carboxylate: Outside the glovebox, *tert*-butyl 4-bromo-1*H*-imidazole-1-carboxylate (123 mg, 0.5 mmol), 4-fluorobenzeneboronic acid (139 mg, 1.0 mmol), and $K_3PO_4$ (159 mg, 1.5 mmol) were weighed into an oven-dried 4-dram vial. The vial was brought into the glovebox, and $^{DIPP}$DAB–Ni–COD (27 mg, 0.05 mmol) and $PCy_3$ (28 mg, 0.1 mmol) were weighed into the vial. Anhydrous 2-MeTHF (3 mL) was added using a 1000 μL micropipette, and the vial was then sealed with a Teflon-lined cap and brought out of the glovebox. The reaction mixture was placed on a tumble stirrer at 80 ºC for 18 h. The solution was then filtered through Celite, dried *in vacuo*, and subsequently dissolved in a minimum amount of toluene. NMR spectroscopy indicated a 46% solution yield relative to a 1,3,5-trimethoxybenzene internal standard. The product was isolated using automated column chromatography (0 → 35% EtOAc/hexanes) in a 34% yield.

$^{1}H$ NMR (300 MHz; $CDCl_3$) δ 8.08 (d,1H), 7.75 (m, 2H), 7.55 (d, 1H), 7.07 (t, 2H), 1.63 (s, 9H).

$^{19}F$ NMR (282 MHz; $CDCl_3$) δ -114.45.

$^{13}C$ NMR (125 MHz; $CDCl_3$) 163.4, 161.5, 147.0, 142.1, 137.2, 129.1, 129.1, 127.0, 127.0, 115.7, 115.5, 111.7, 85.8, 27.9.

HR-MS (ESI) theoretical ($C_{14}H_{16}N_2O_2F$) $[M+H]^+$ *m/z* 263.11903; found *m/z* 263.11899.

## V. Solubility and Stability of Nickel Complexes

The solubility of $Ni(COD)_2$, Ni(COD)(DQ), $^{DIPP}$DAB–Ni–COD, $^{DMP}$DAB–Ni–COD, $^{DIPP}$DAB–Ni–FN, and $^{DMP}$DAB–Ni–FN was evaluated by weighing 20 mg of each complex into an oven-dried 1-dram vial under inert atmosphere. 1,3,5-Trimethoxybenzene was weighed into each vial, and 1.0 mL of the selected anhydrous solvent was added using a 1000 μL micropipette. The solutions were stirred for 5 minutes prior to filtration through a small Celite plug into a screw-cap NMR tube. $^{1}H$ NMR spectra were taken with a delay time of 30 seconds to quantify the solubility.

The stability of the aforementioned complexes in each solvent was evaluated by taking NMR spectra every hour for the following 4-6 hours, as well as 24 hours and 48 hours after the initial solution preparation. As the complexes decompose over time, the peaks in the NMR spectrum broaden due to the presence of trace paramagnetic impurities.

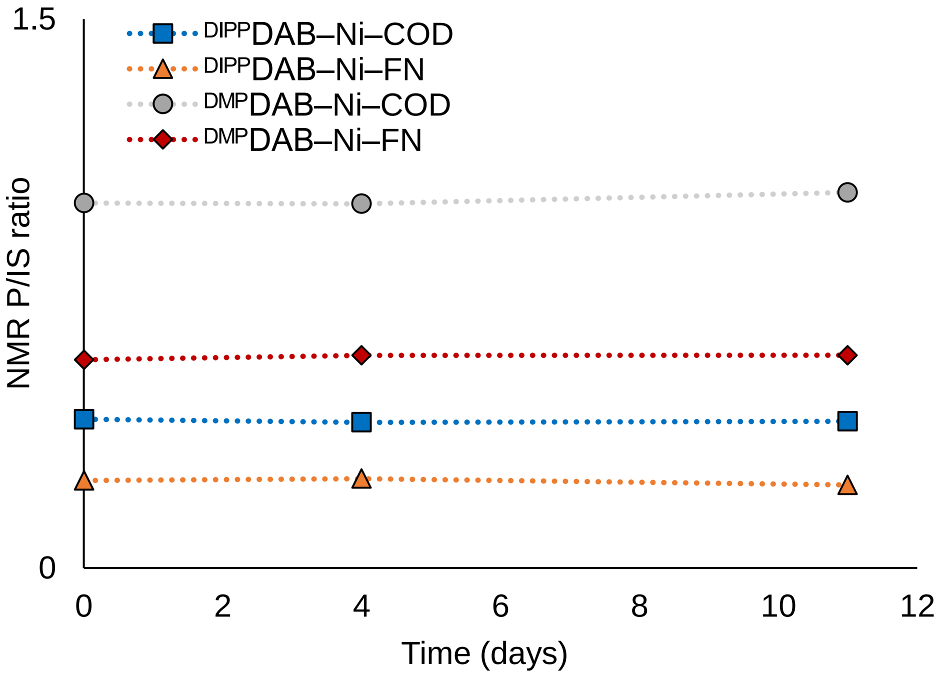


**Figure S2**. Solution stability of complexes **1-4** at 20 mg/mL in $C_6D_6$ under inert atmosphere using $^{1}H$ NMR spectroscopy.

**$^{DIPP}$DAB–Ni–COD**

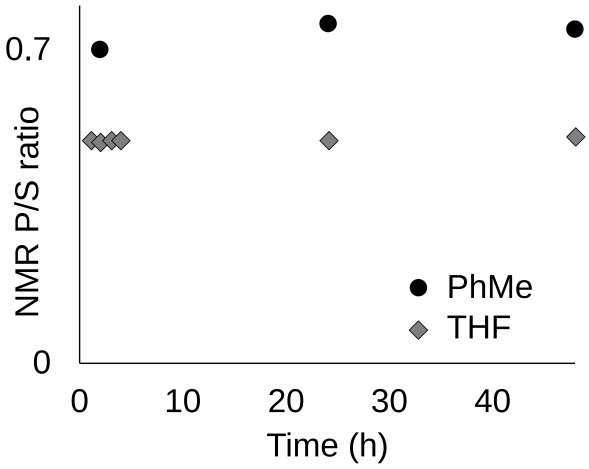


**Figure S3**. Solution stability of $^{DIPP}$DAB–Ni–COD at 20 mg/mL (initial charge) in two deuterated solvents under inert atmosphere using $^{1}$H NMR spectroscopy.

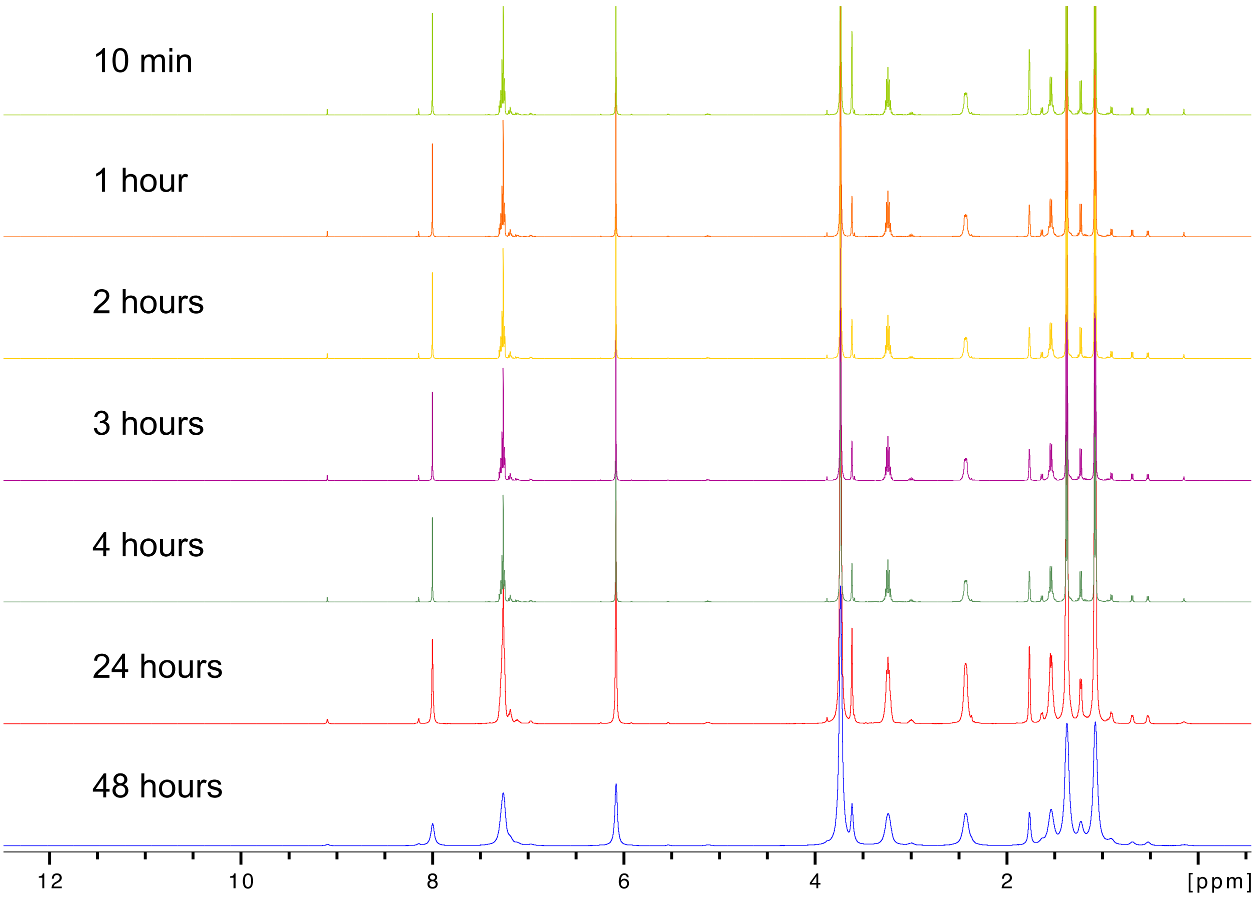


**Figure S4.** Stability of $^{DIPP}$DAB–Ni–COD in $d_8$-THF over time at room temperature under inert atmosphere. Sample contains 1,3,5-trimethoxybenzene.

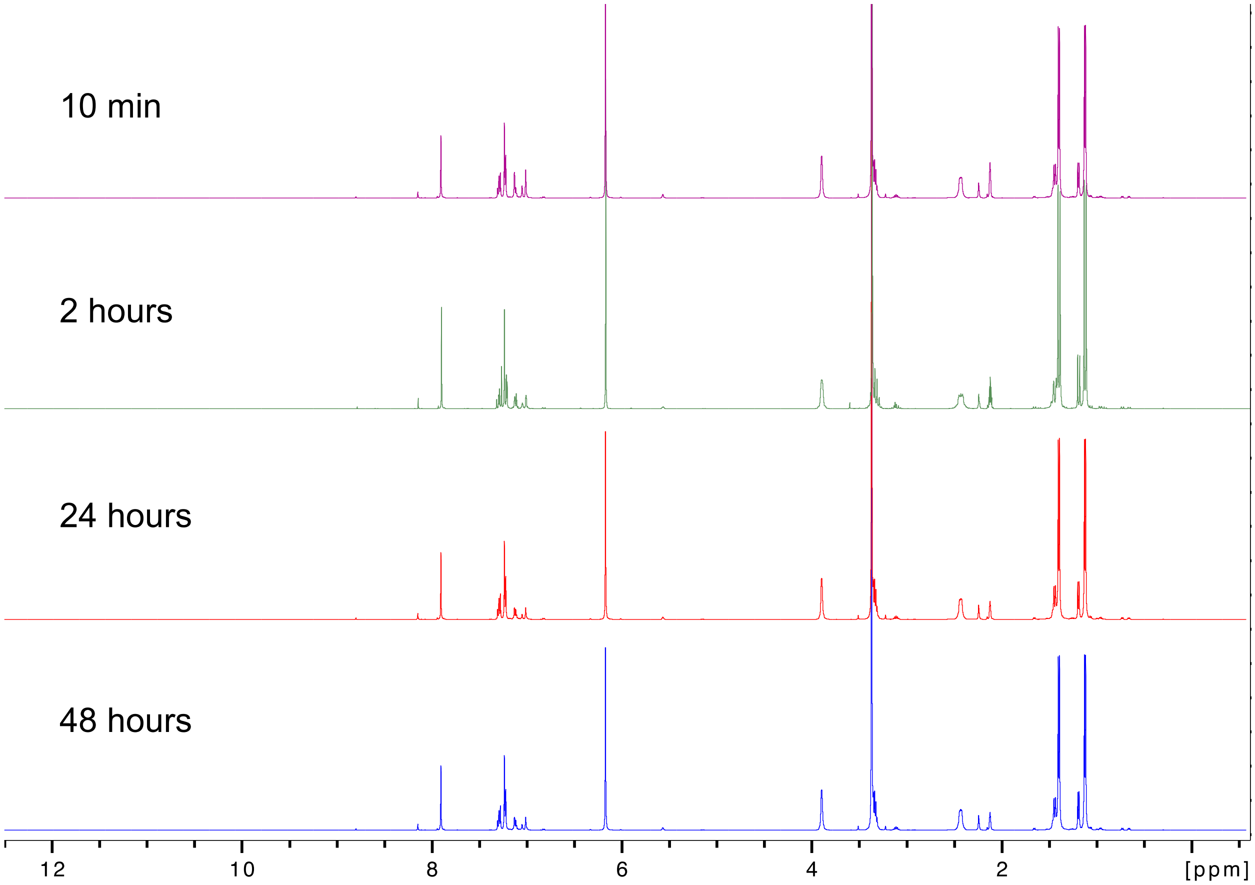


**Figure S5.** Stability of $^{DIPP}$DAB–Ni–COD in d$_8$-toluene over time at room temperature under inert atmosphere. Sample contains 1,3,5-trimethoxybenzene.

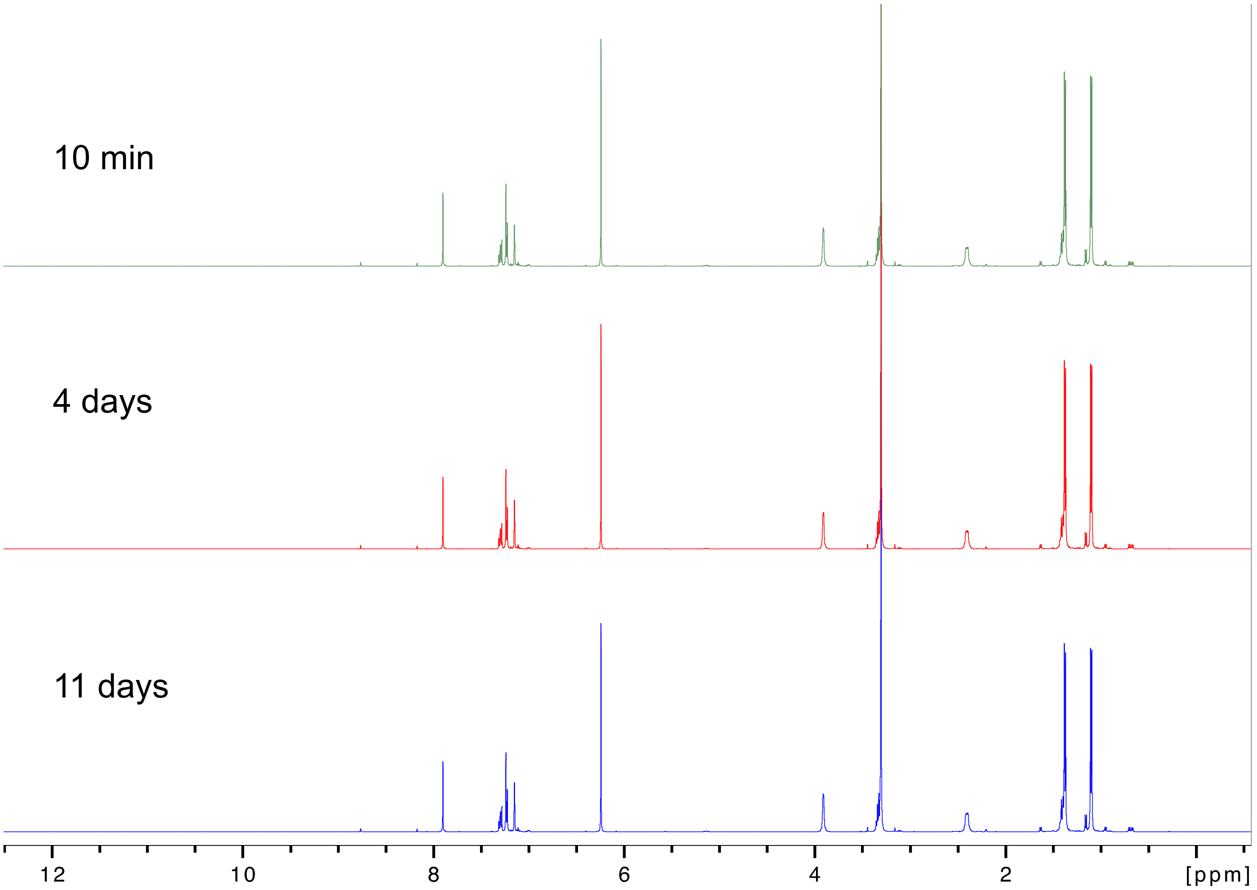


**Figure S6.** Stability of $^{DIPP}$DAB–Ni–COD in $C_6D_6$ over time at room temperature under inert atmosphere. Sample contains 1,3,5-trimethoxybenzene.

***<u>Air stability evaluation of solid $^{DIPP}$DAB–Ni–COD:</u>***

Outside the glovebox, 1,3,5-trimethoxybenzene was weighed into two separate 1-dram vials. One vial was brought into the glovebox, and $^{DIPP}$DAB–Ni–COD (14 mg, 0.026 mmol) was added. A second portion of $^{DIPP}$DAB–Ni–COD was removed from the glovebox, and was weighed into the 1-dram vial containing internal standard in open air (16 mg, 0.029 mmol). After weighing the solid, the vial was brought into the glovebox. 1 mL of $C_6D_6$ was added to both vials containing $^{DIPP}$DAB–Ni–COD and internal standard. These solutions were mixed to ensure complete dissolution, and then filtered through a Celite plug in a Pasteur pipette directly into screw-cap NMR tubes. The amount of intact $^{DIPP}$DAB–Ni–COD in each sample was measured using $^{1}$H NMR spectroscopy.

**Table S1.** Air stability of solid $^{DIPP}$DAB–Ni–COD.[a]

| | **mmol weighed** | **mmol calculated**[b] |
|---|---|---|
| Glovebox | 0.026 | 0.026 |
| Air | 0.029 | 0.025 |

[a]The same batch of $^{DIPP}$DAB–Ni–COD was used to evaluate the air stability under inert atmosphere vs air to prevent potential batch-to-batch discrepancies. [b]Measured using $^{1}$H NMR spectroscopy with 1,3,5-trimethoxybenzene as internal standard.

**$^{DMP}$DAB–Ni–COD**

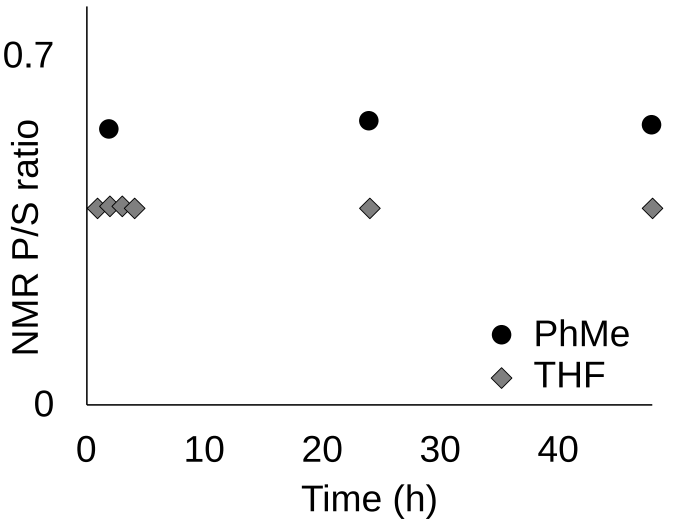


**Figure S7.** Solution stability of $^{DMP}$DAB–Ni–COD at 20 mg/mL (initial charge) in two deuterated solvents under inert atmosphere using $^{1}$H NMR spectroscopy

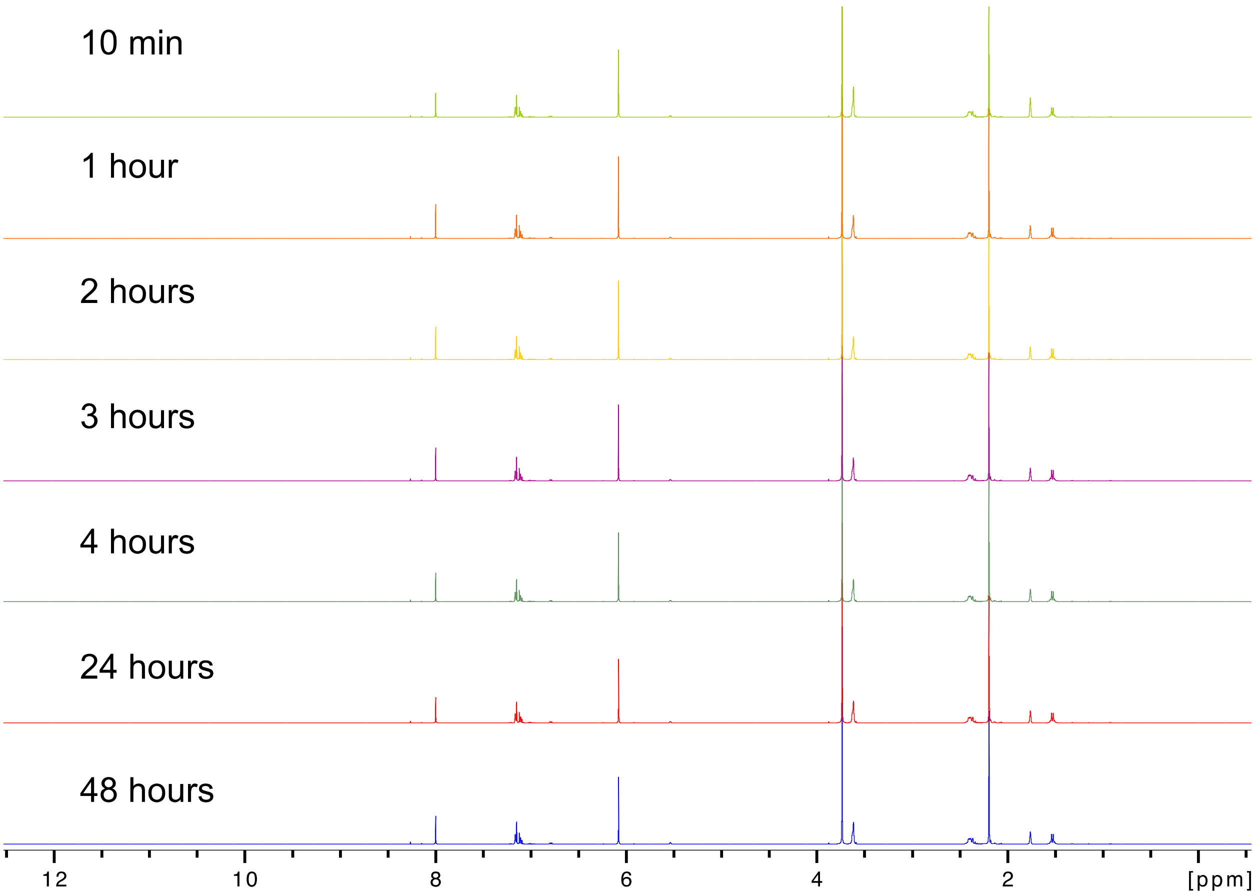


**Figure S8.** Stability of $^{DMP}$DAB–Ni–COD in $d_8$-THF over time at room temperature under inert atmosphere. Sample contains 1,3,5-trimethoxybenzene.

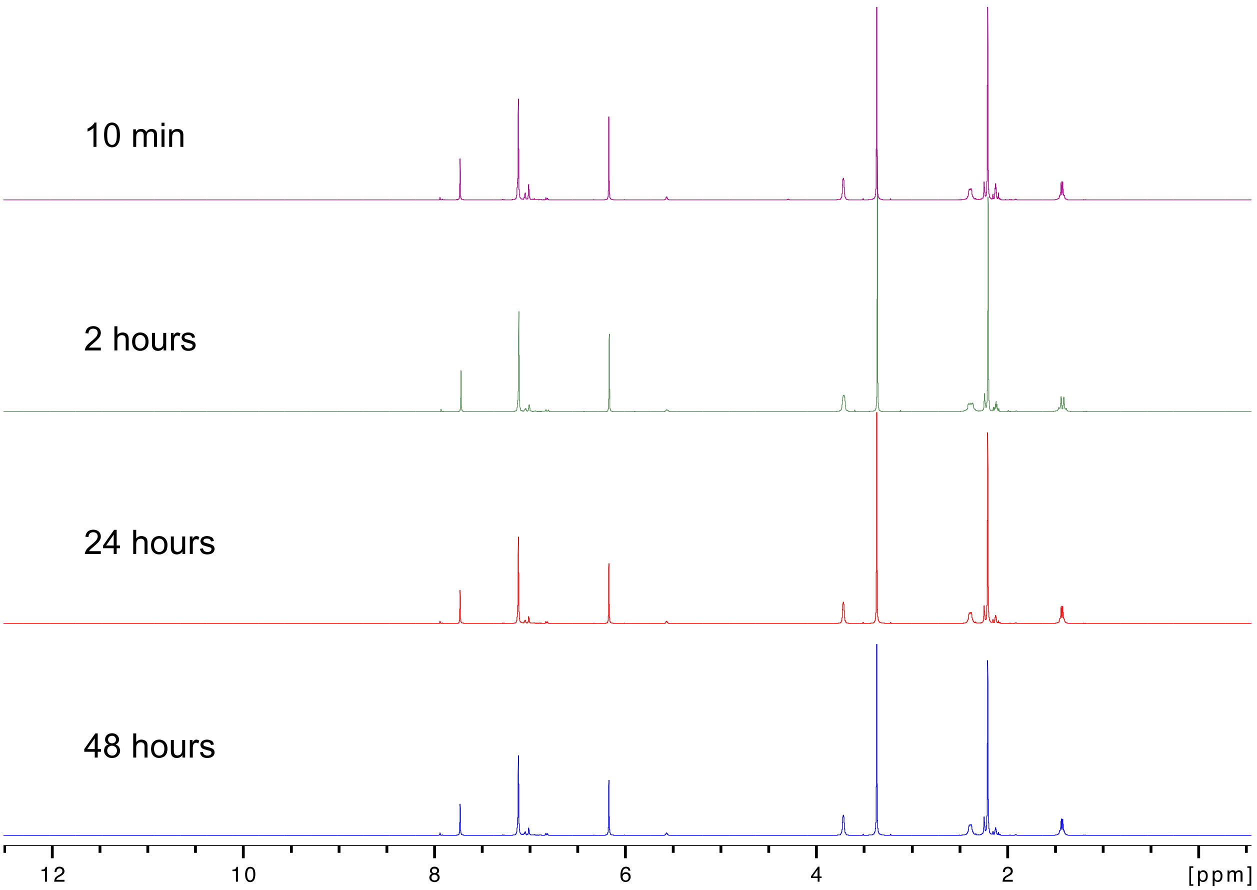


**Figure S9.** Stability of $^{DMP}$DAB–Ni–COD in $d_8$-toluene over time at room temperature under inert atmosphere. Sample contains 1,3,5-trimethoxybenzene.

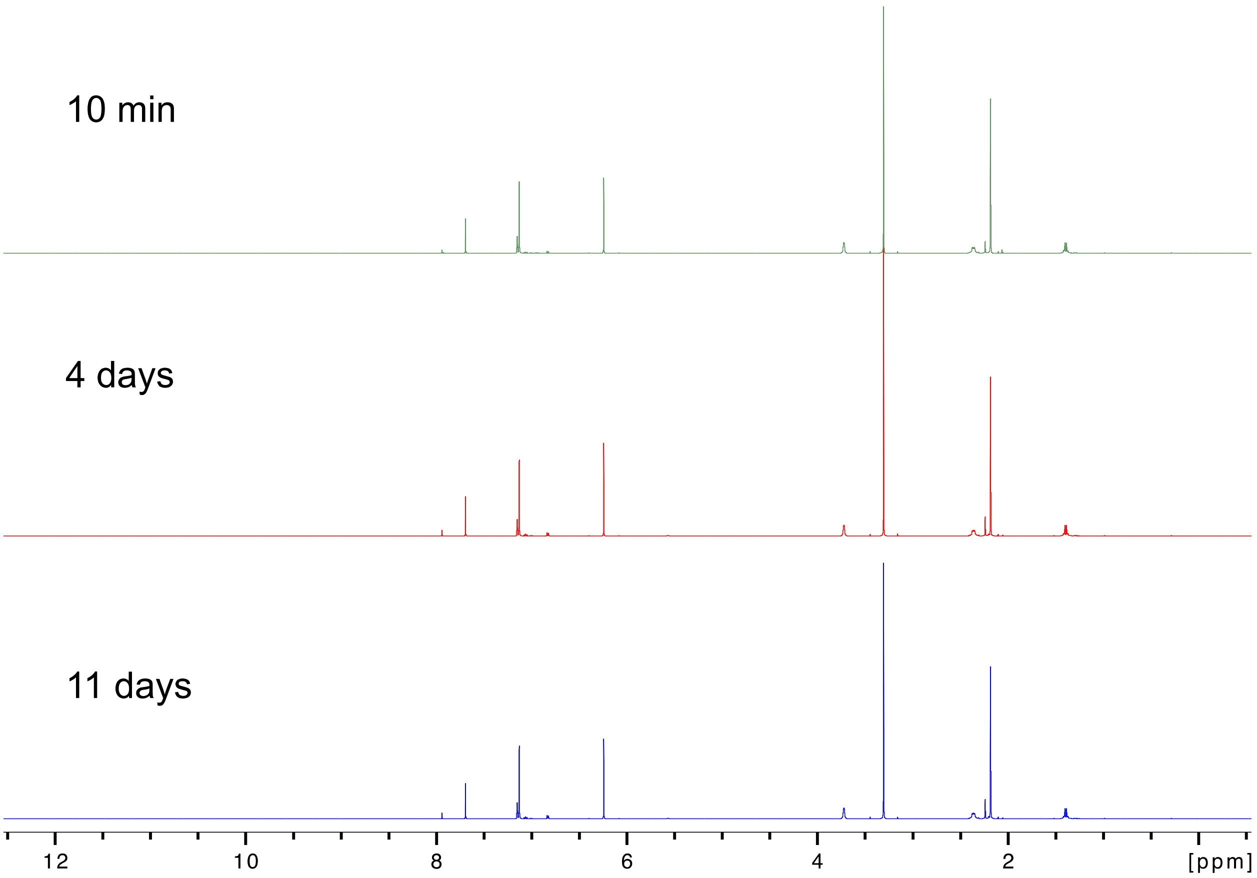


**Figure S10.** Stability of $^{DMP}$DAB–Ni–COD in $C_6D_6$ over time at room temperature under inert atmosphere. Sample contains 1,3,5-trimethoxybenzene.

### $^{DIPP}$DAB–Ni–FN

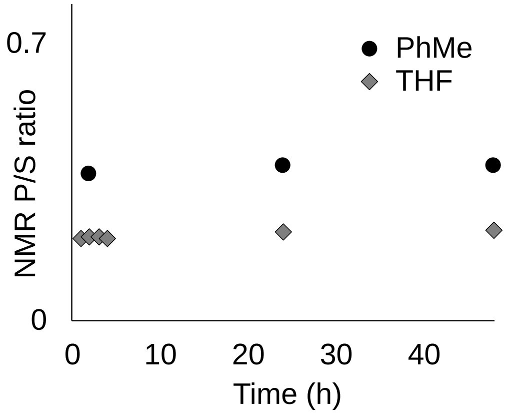


**Figure S11.** Solution stability of $^{DIPP}$DAB–Ni–FN at 20 mg/mL (initial charge) in two deuterated solvents under inert atmosphere using $^{1}$H NMR spectroscopy.

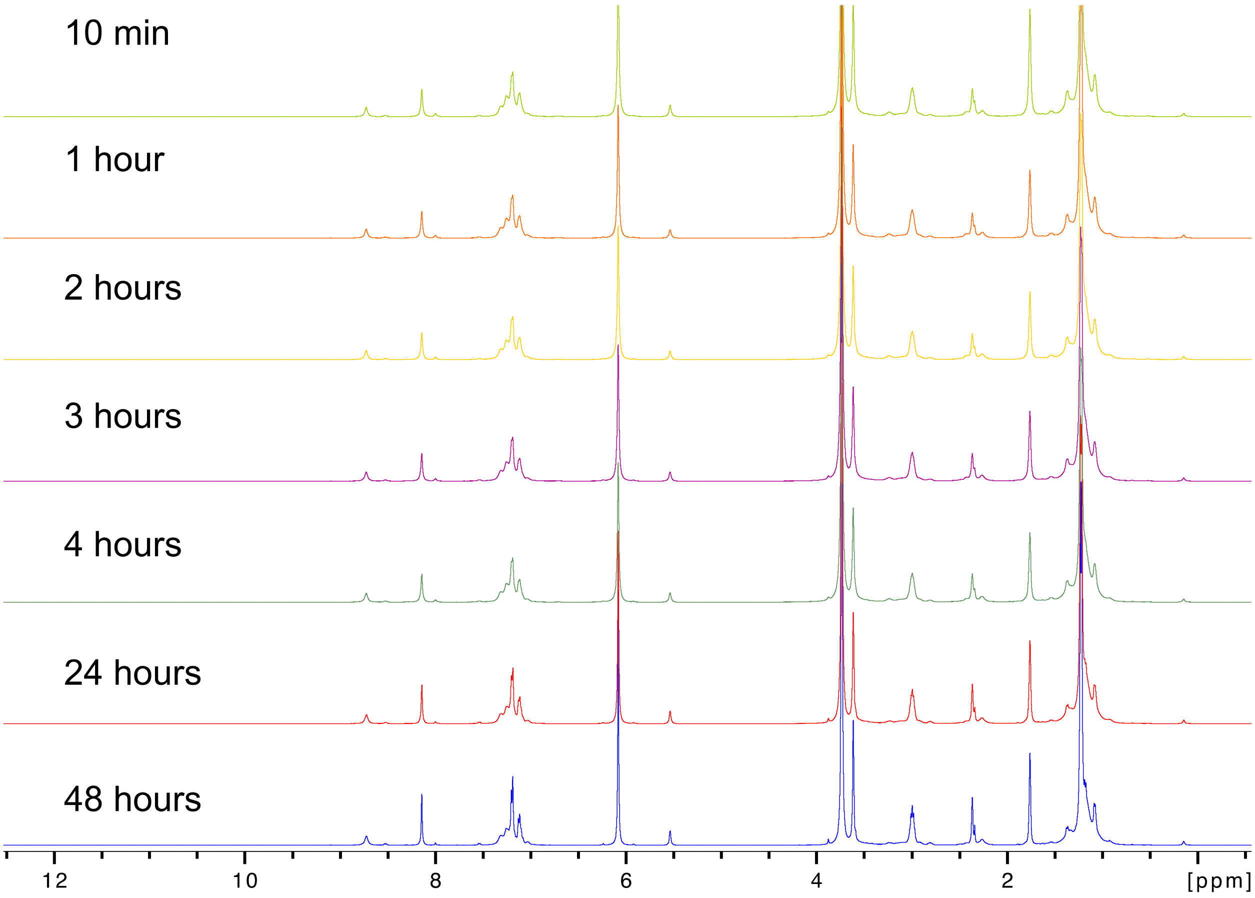


**Figure S12.** Stability of $^{DIPP}$DAB–Ni–FN in $d_8$-THF over time at room temperature under inert atmosphere. Sample contains 1,3,5-trimethoxybenzene.

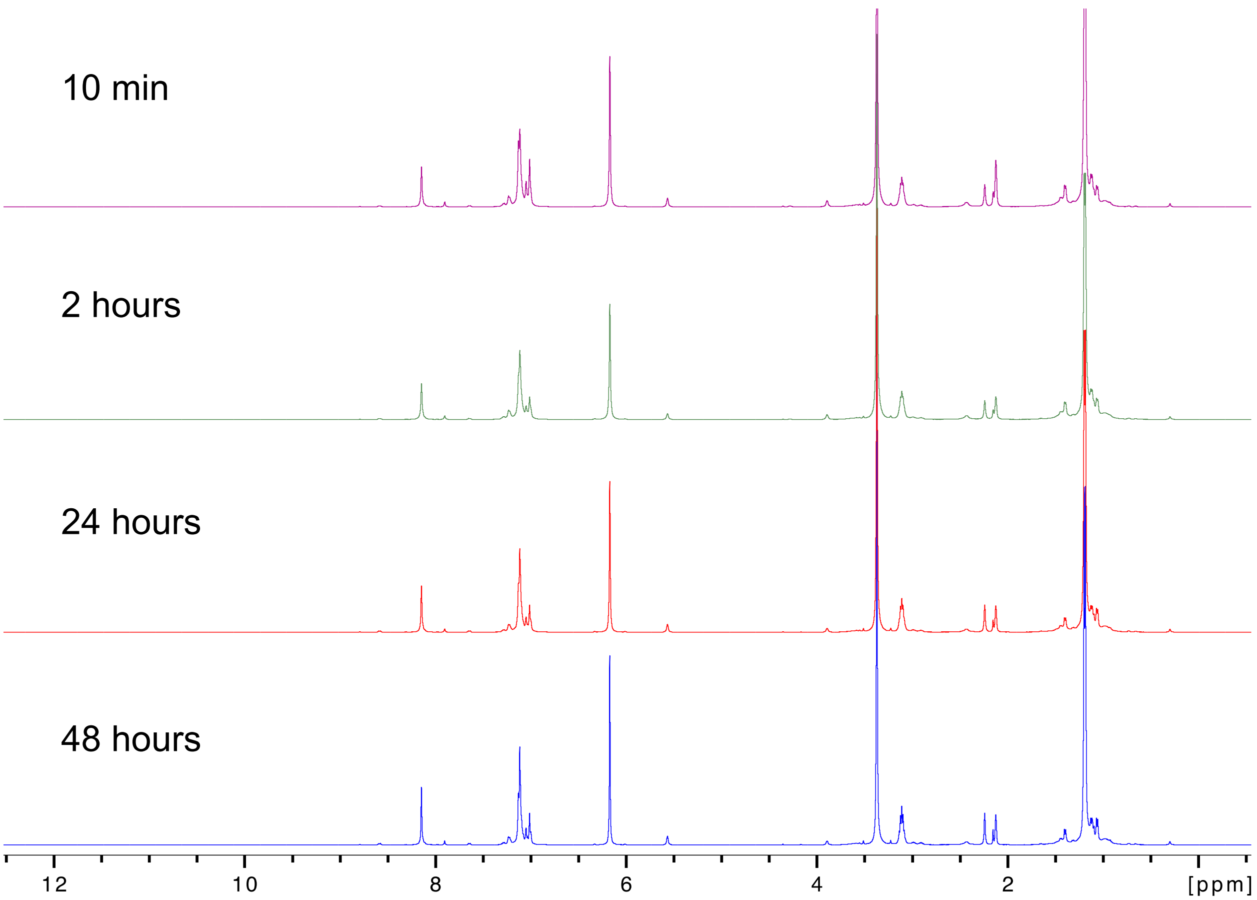


**Figure S13.** Stability of $^{DIPP}$DAB–Ni–FN in $d_8$-toluene over time at room temperature under inert atmosphere. Sample contains 1,3,5-trimethoxybenzene.

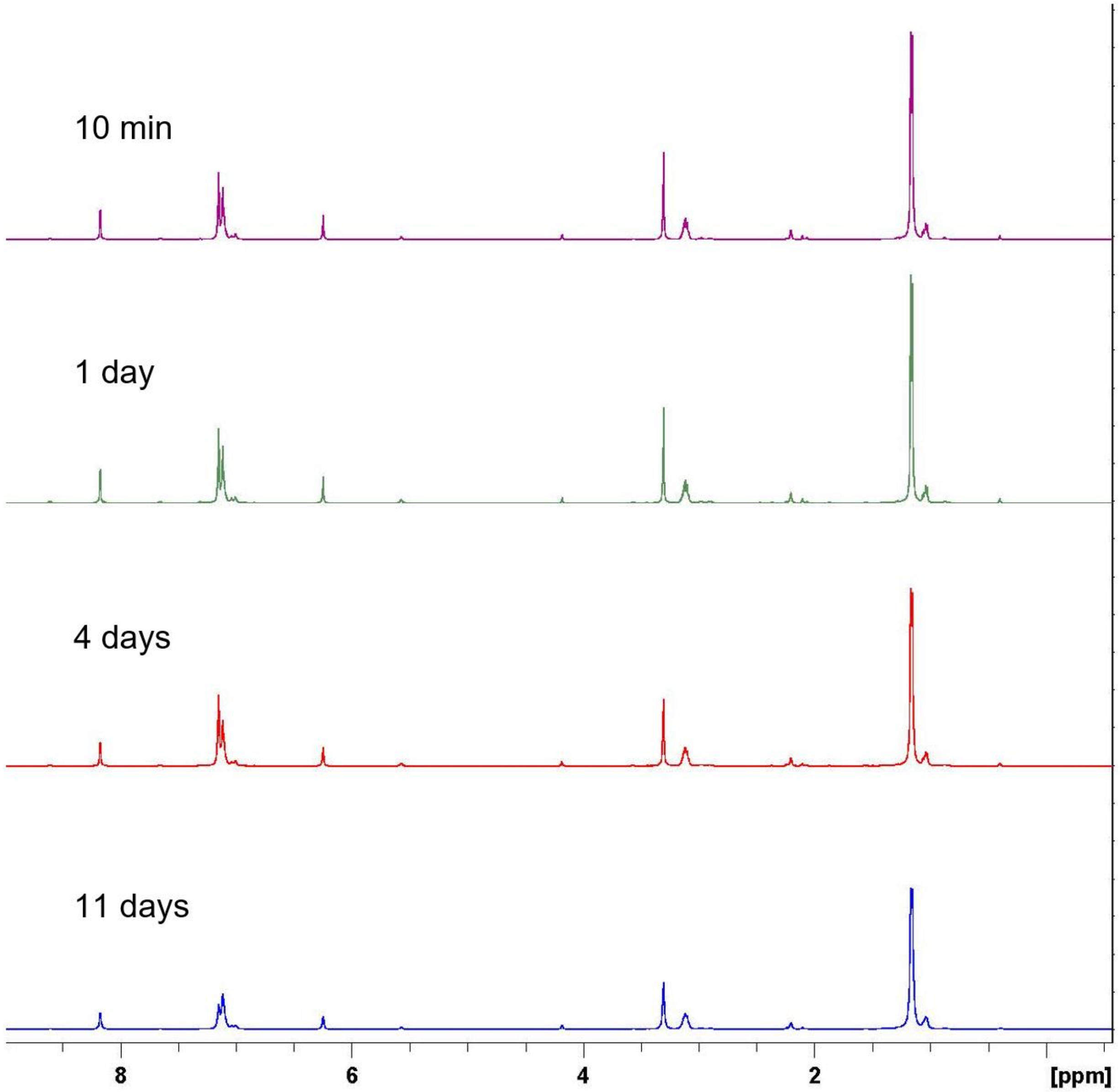


**Figure S14.** Stability of $^{DIPP}$DAB–Ni–FN in $C_6D_6$ over time at room temperature under inert atmosphere. Sample contains 1,3,5-trimethoxybenzene.

## $^{DMP}$DAB–Ni–FN

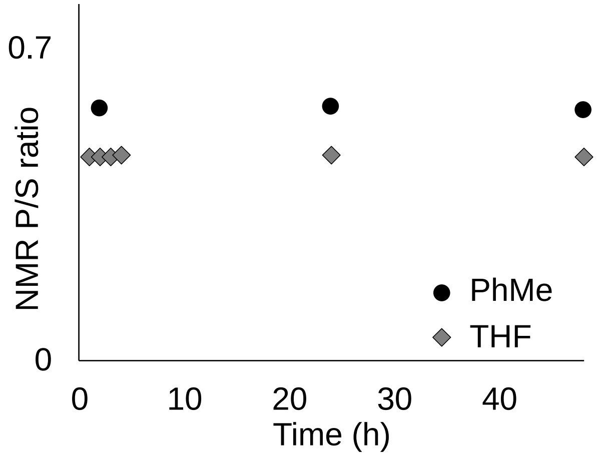


**Figure S15.** Solution stability of $^{DMP}$DAB–Ni–FN at 20 mg/mL in two deuterated solvents under inert atmosphere using $^{1}$H NMR spectroscopy.

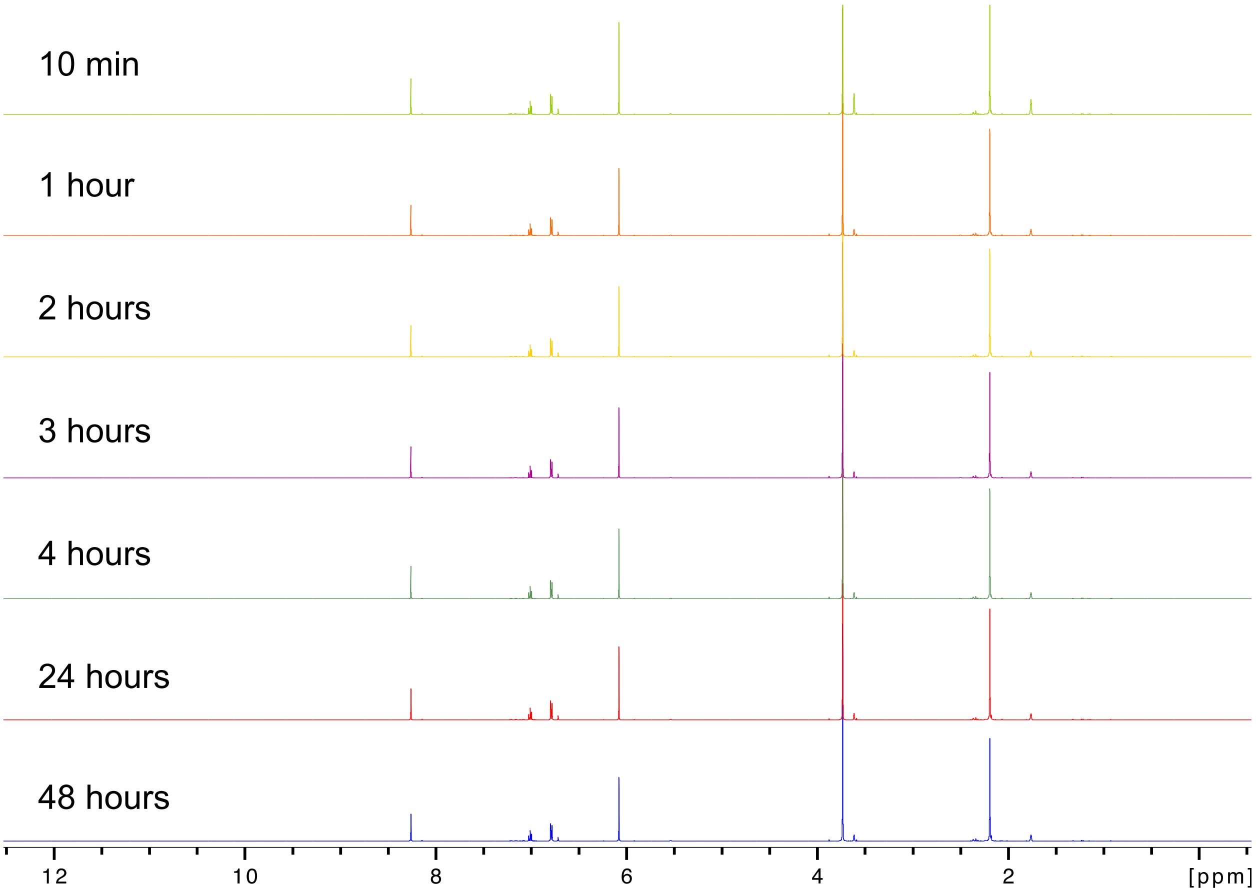


**Figure S16.** Stability of $^{DMP}$DAB–Ni–FN in $d_8$-THF over time at room temperature under inert atmosphere. Sample contains 1,3,5-trimethoxybenzene.

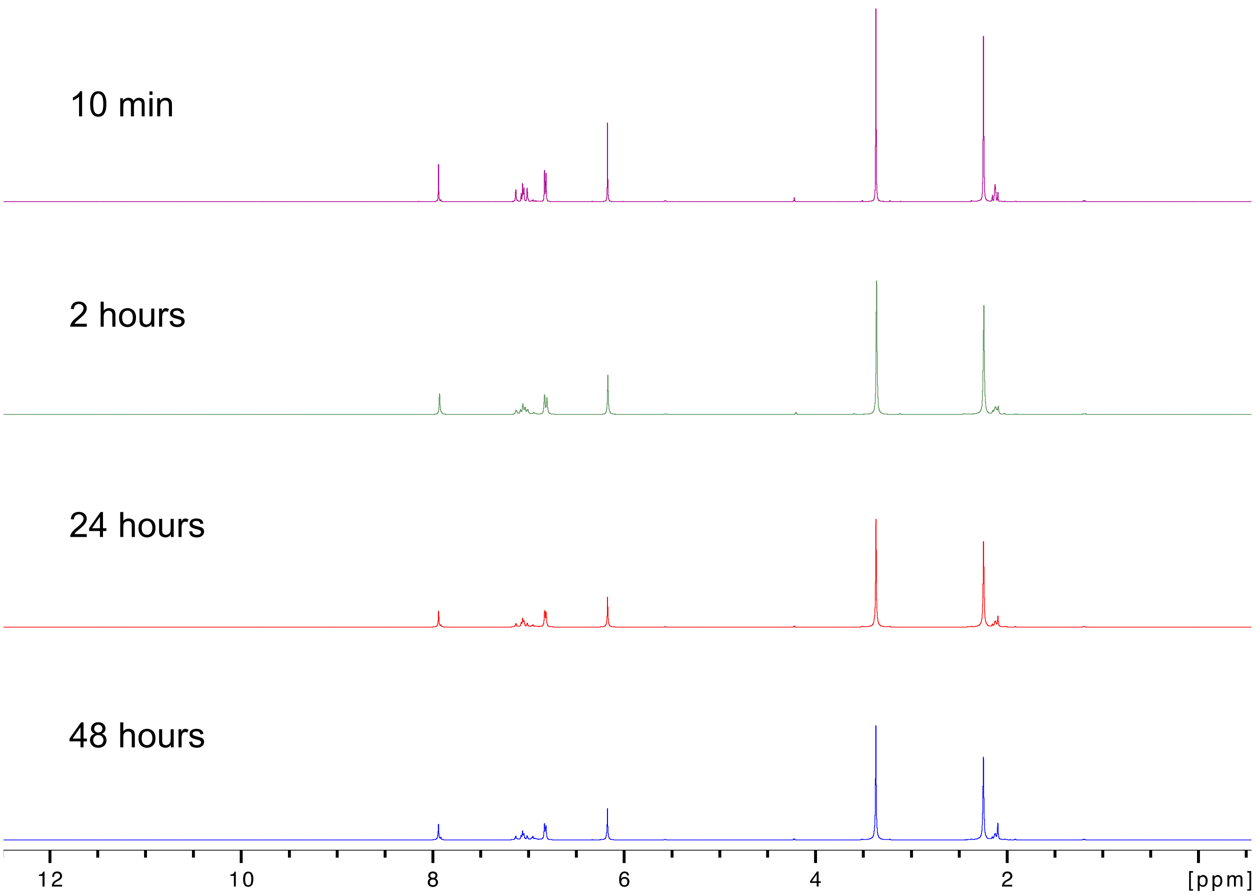


**Figure S17.** Stability of $^{DMP}$DAB–Ni–FN in $d_8$-toluene over time at room temperature under inert atmosphere. Sample contains 1,3,5-trimethoxybenzene.

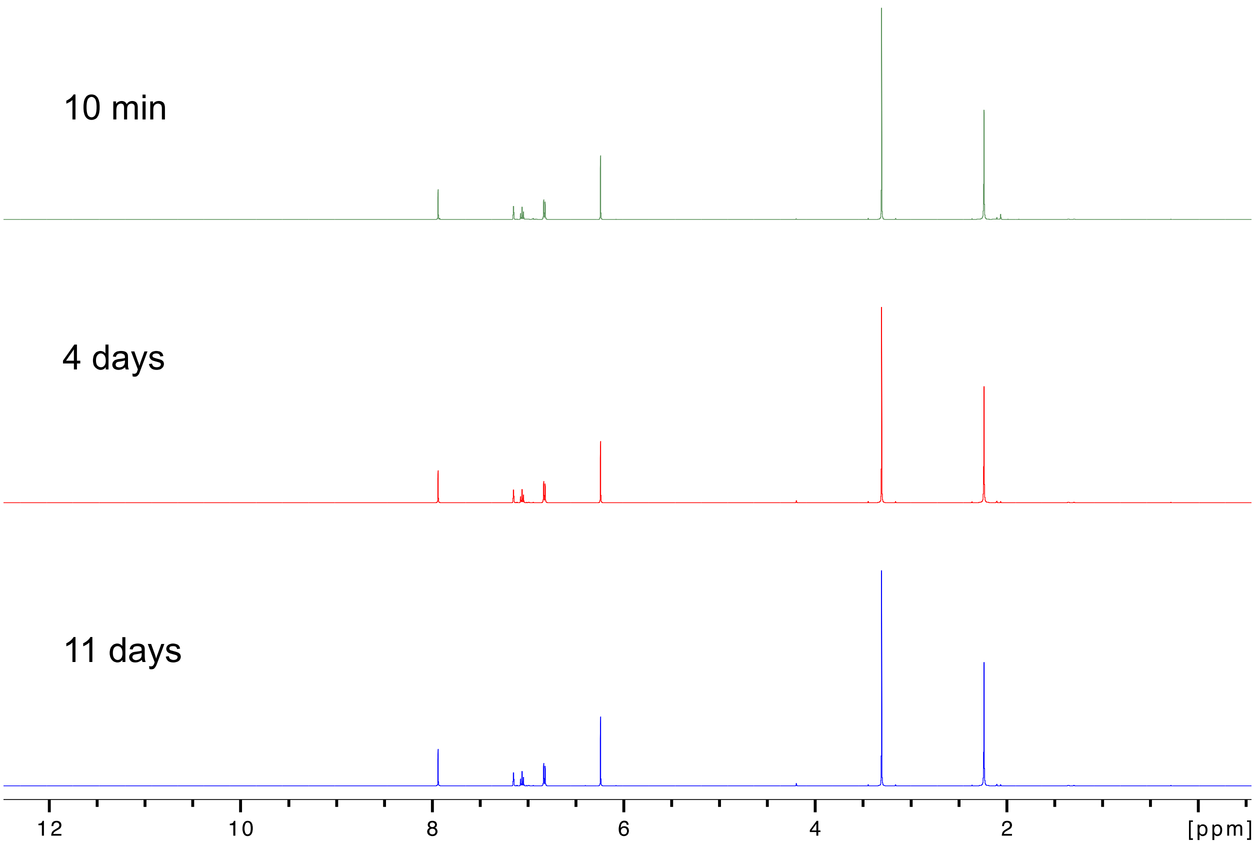

**Figure S18.** Stability of $^{DMP}$DAB–Ni–FN in $C_6D_6$ over time at room temperature under inert atmosphere. Sample contains 1,3,5-trimethoxybenzene.

## VI. Ligand exchange reaction monitoring experiment

Inside the glovebox, 13.8 mg of the $Ni(COD)_2$ catalyst (1 equiv, 0.05 mmol) and $^{DMP}$DAB (1 equiv, 13.2 mg) were weighed into a 1 dram vial. $d_8$-THF was added (0.5 mL) and the solution was filtered through a plug of Celite. Fumaronitrile (1 equiv, 3.9 mg) was dissolved in 0.2 mL of $d_8$-THF and filtered through the same Celite plug. The resulting solution was analyzed by $^1$H NMR spectroscopy immediately, and after 42 h.

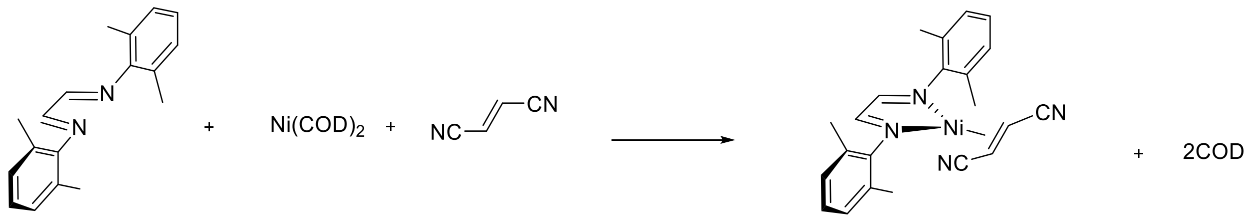


$^{DMP}$DAB-Ni-FN

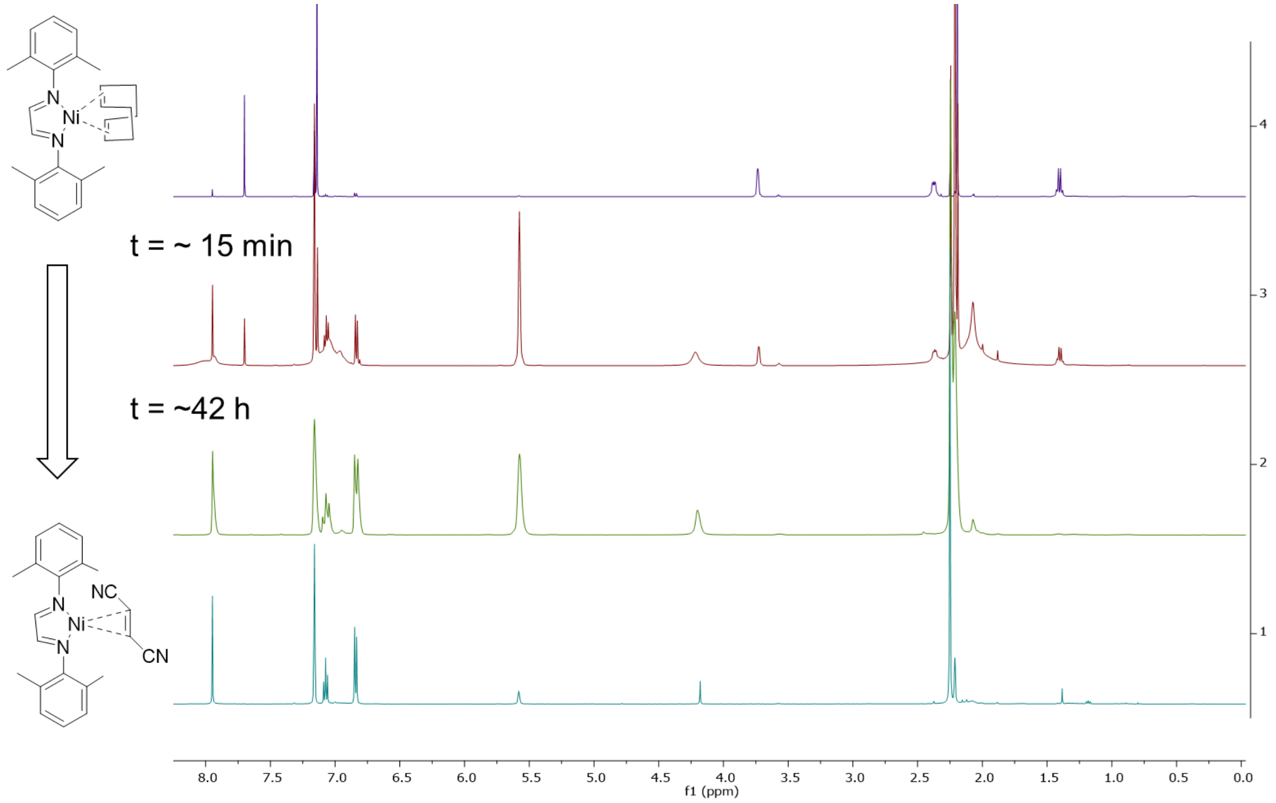

Comparison of the aromatic region of the $^{1}H$ NMR spectra (500 MHz, $C_6D_6$) for free $^{DMP}$DAB, $^{DMP}$DAB-Ni-COD, and $^{DMP}$DAB-Ni-FN

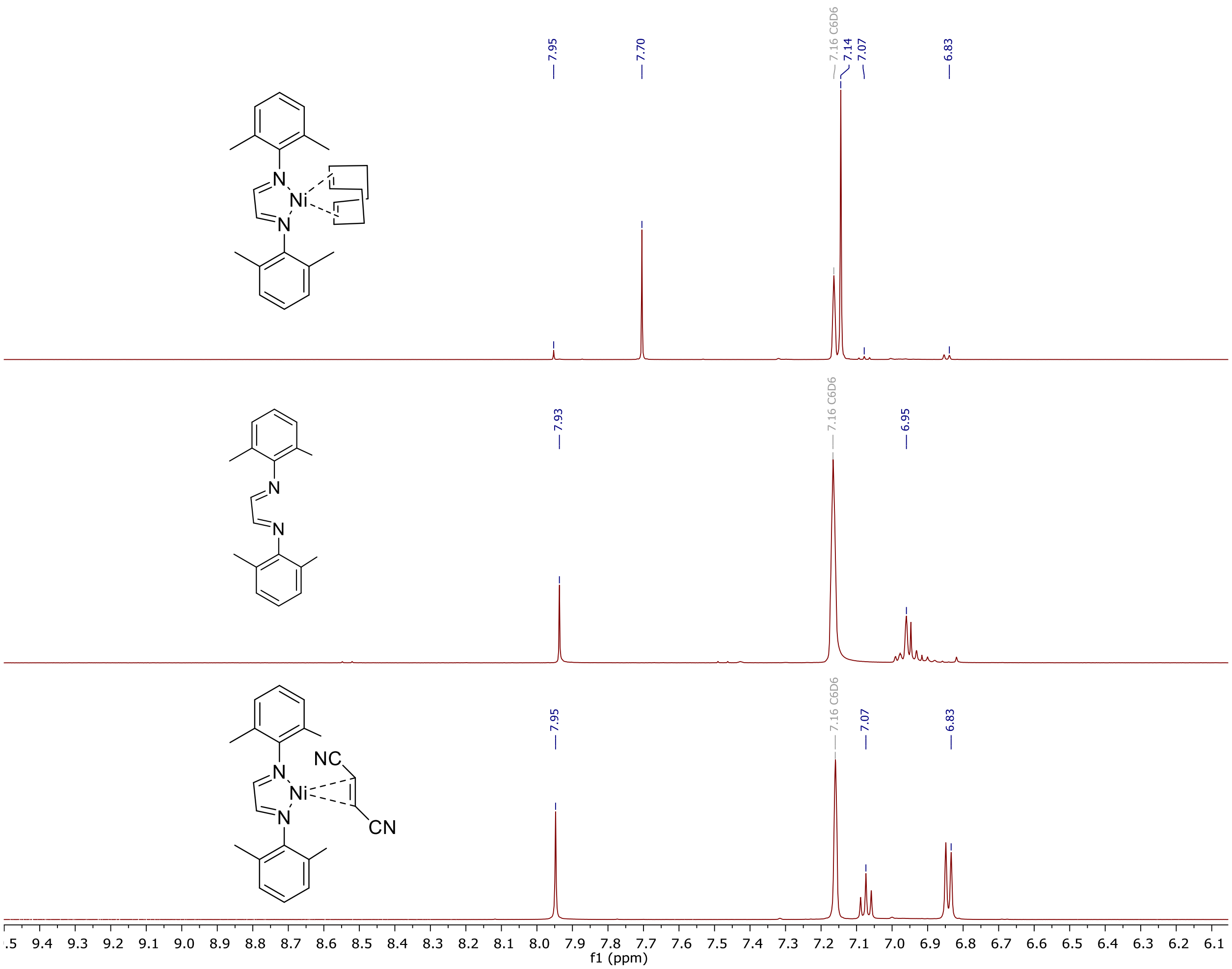

## VII. Spectra

$^{DIPP}$DAB–Ni–COD **1**, $^1$H, 500 MHz, $C_6D_6$

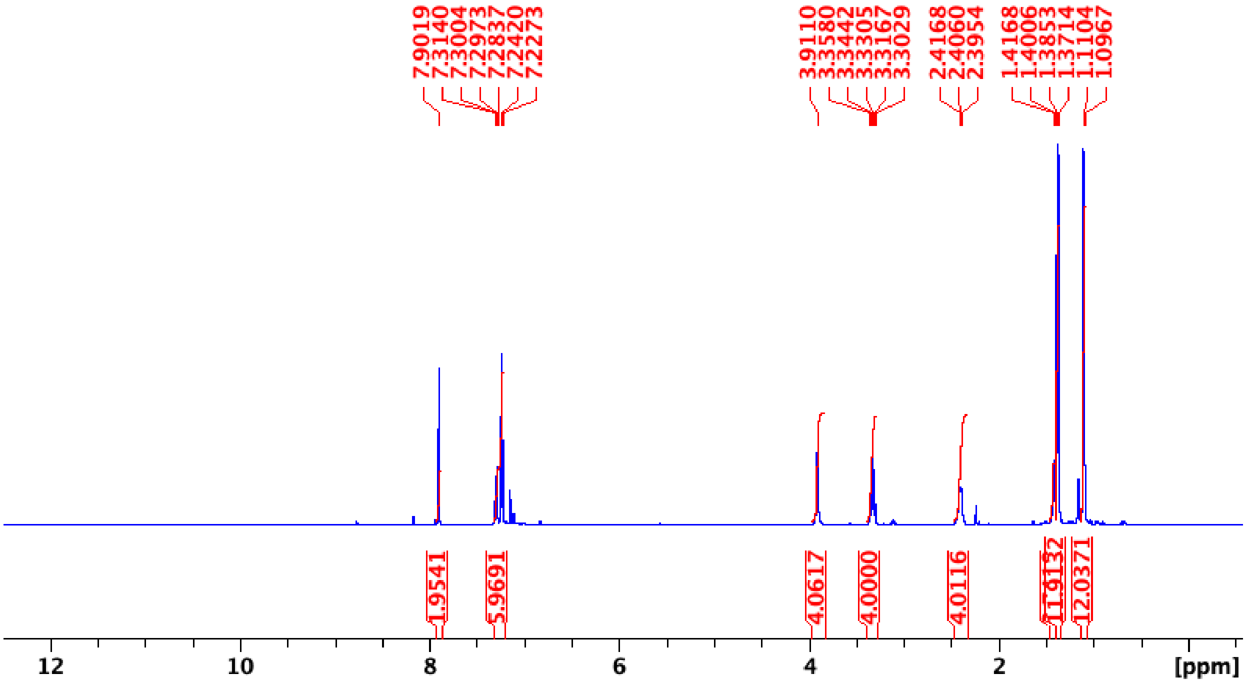


$^{DIPP}$DAB–Ni–COD **1**, $^{13}$C{$^1$H}, 125 MHz, $C_6D_6$

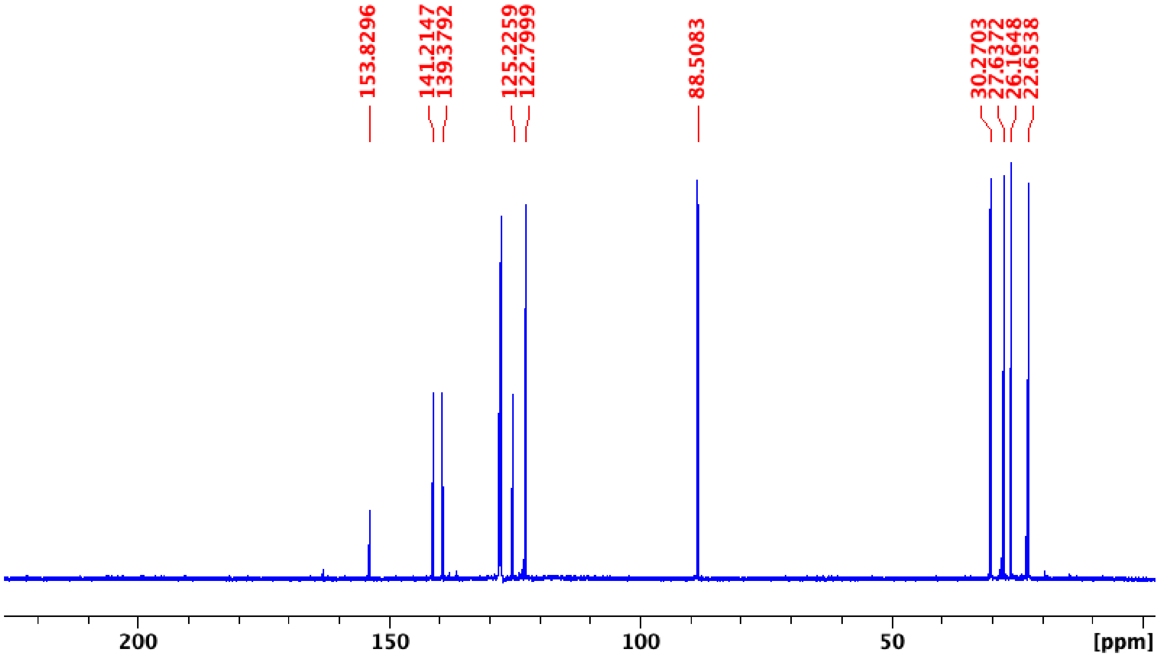

$^{DIPP}$DAB–Ni–COD **1**, $^{1}H$ $^{13}C$ HSQC, $C_6D_6$

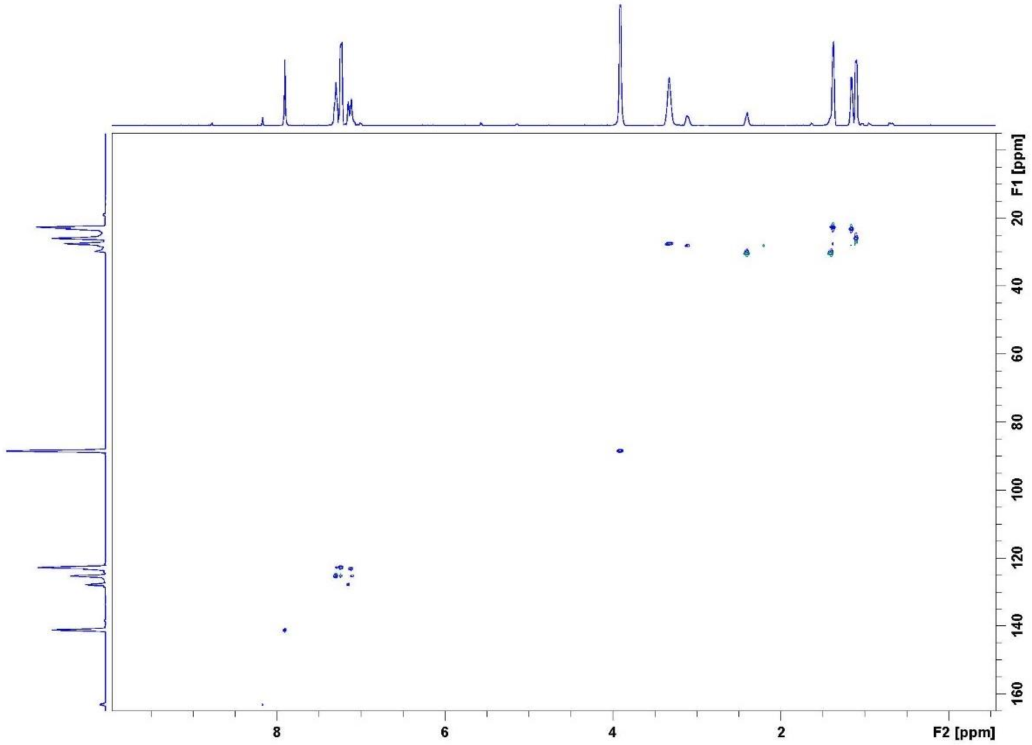


$^{DIPP}$DAB–Ni–COD **1**, $^{1}H$ $^{13}C$ HMBC, $C_6D_6$

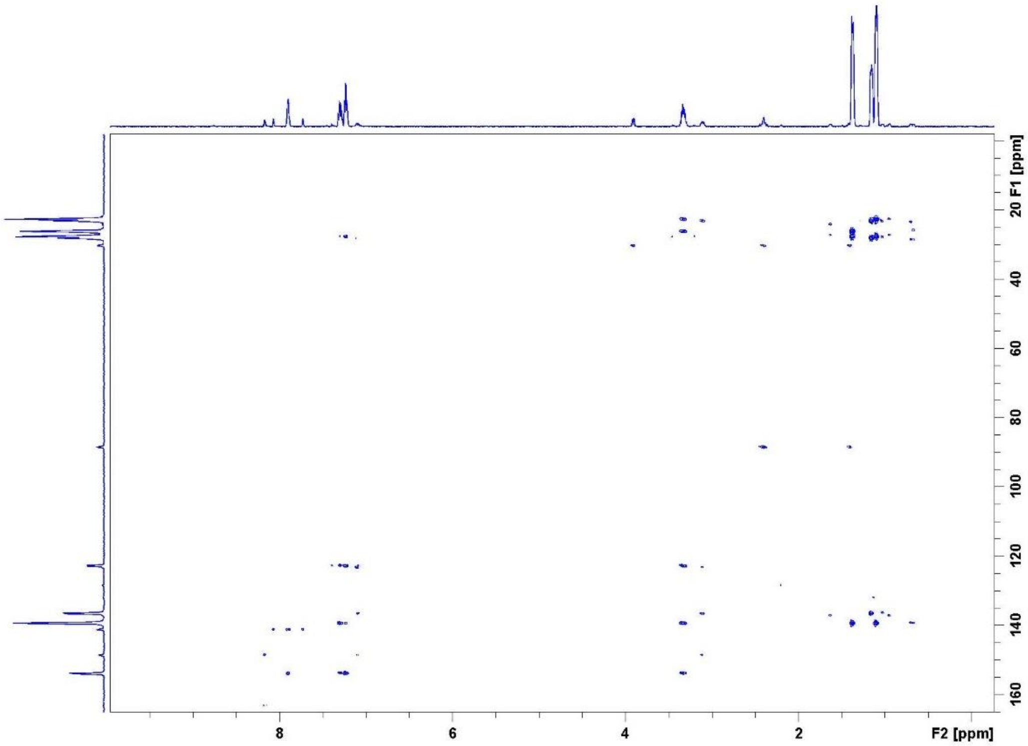

$^{DIPP}$DAB–Ni–COD **1**, HR-MS

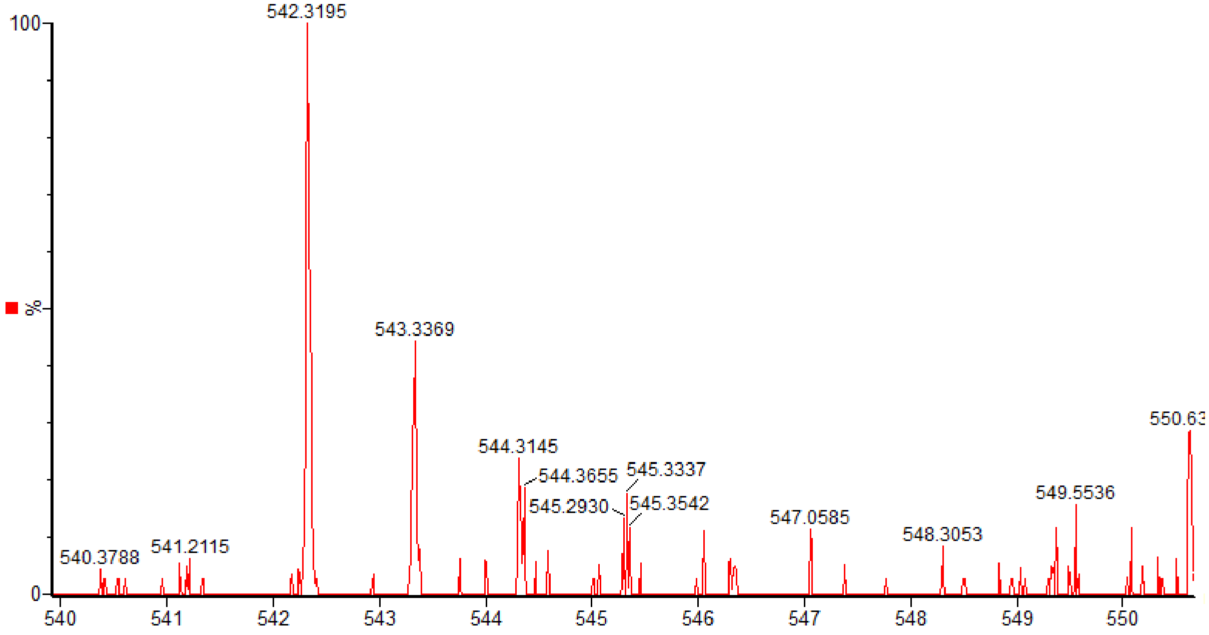


$^{DIPP}$DAB–Ni–COD **1**, IR

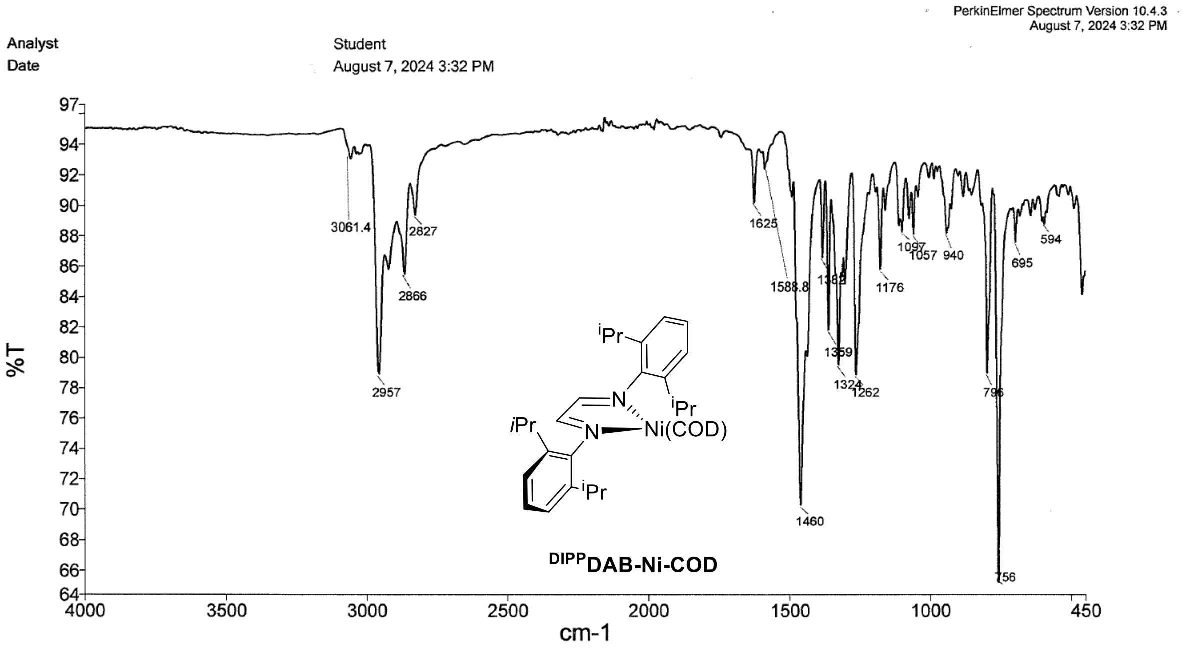

$^{DMP}$DAB–Ni–COD **2**, $^{1}H$, 500 MHz, $C_6D_6$

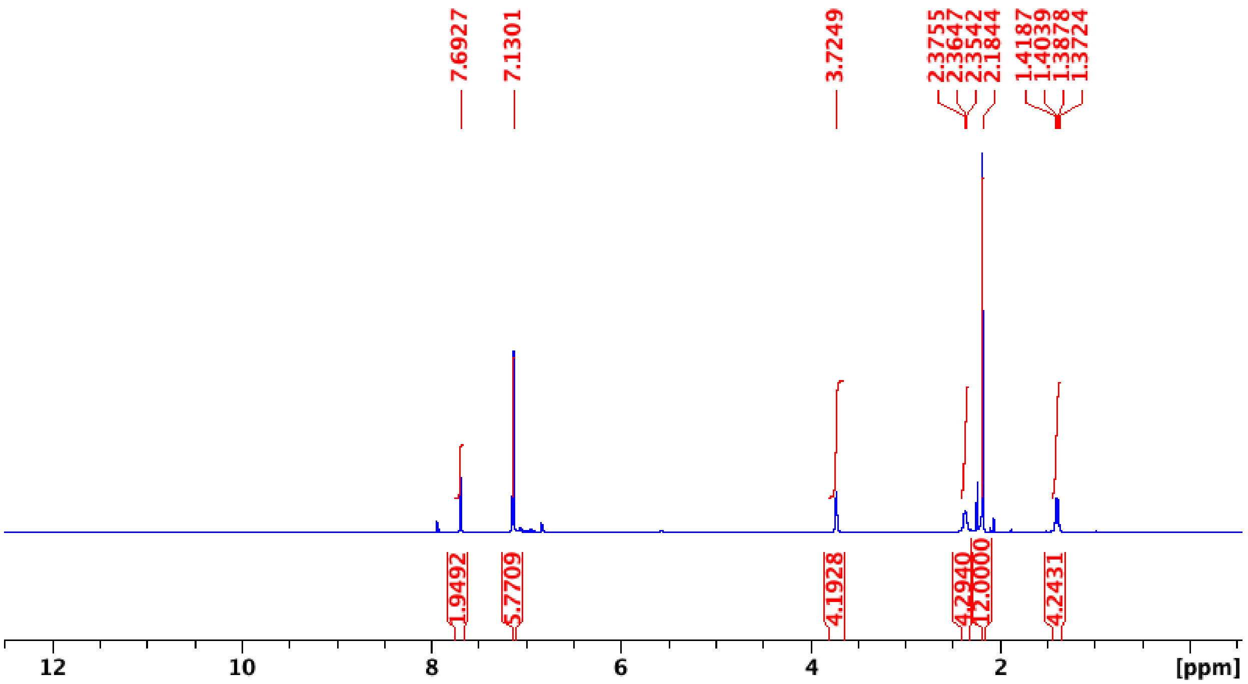


$^{DMP}$DAB–Ni–COD **2**, $^{13}C\{^{1}H\}$, 125 MHz, $C_6D_6$

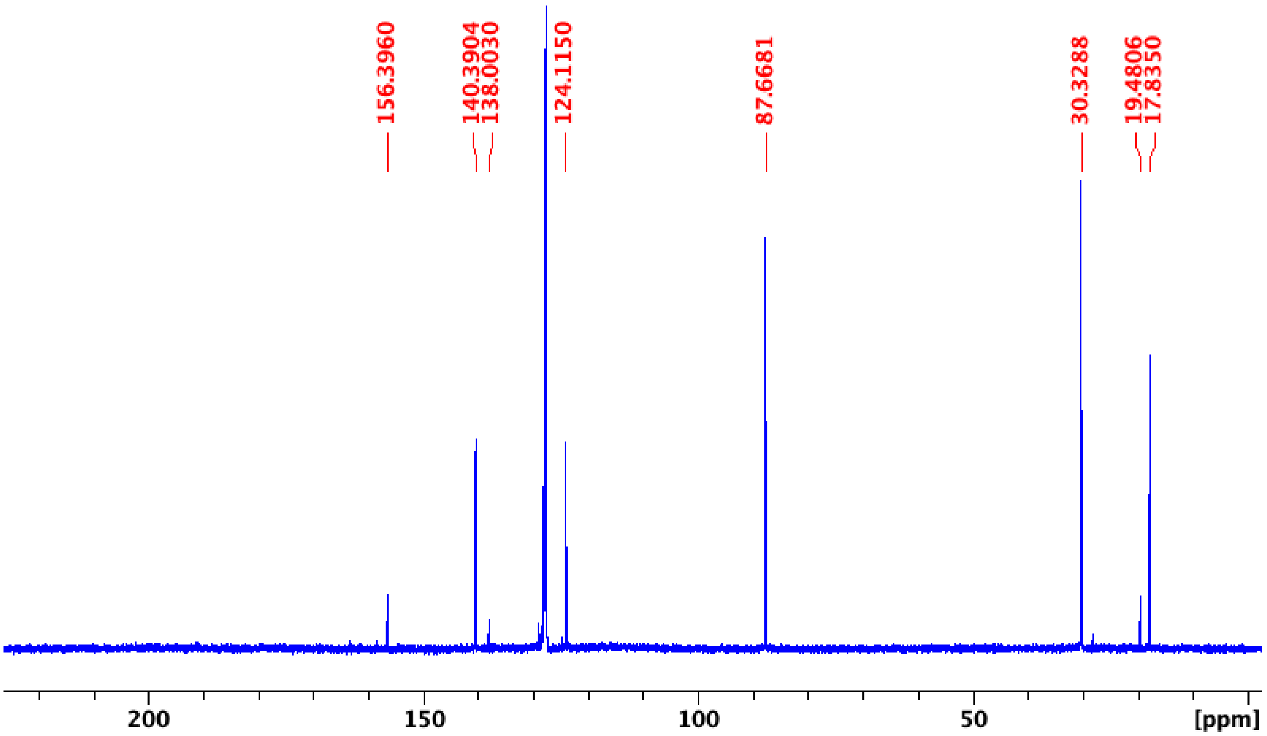

$^{DMP}$DAB–Ni–COD **2**, $^{1}H$ $^{13}C$ HSQC, $C_6D_6$

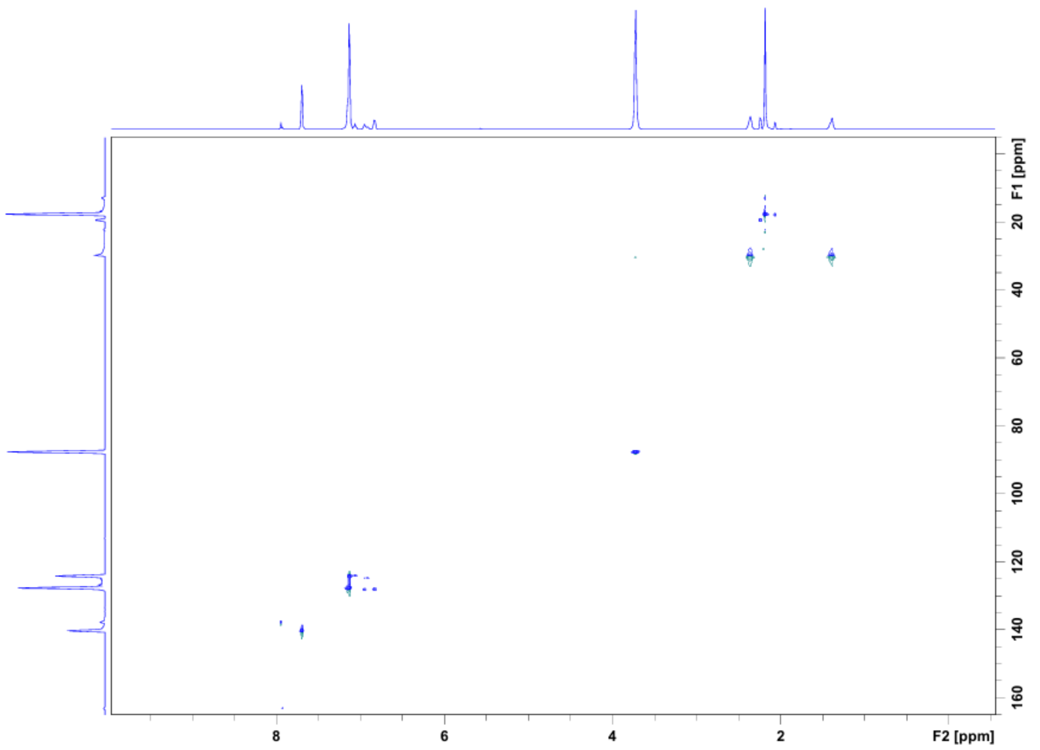


$^{DMP}$DAB–Ni–COD **2**, $^{1}H$ $^{13}C$ HMBC, $C_6D_6$

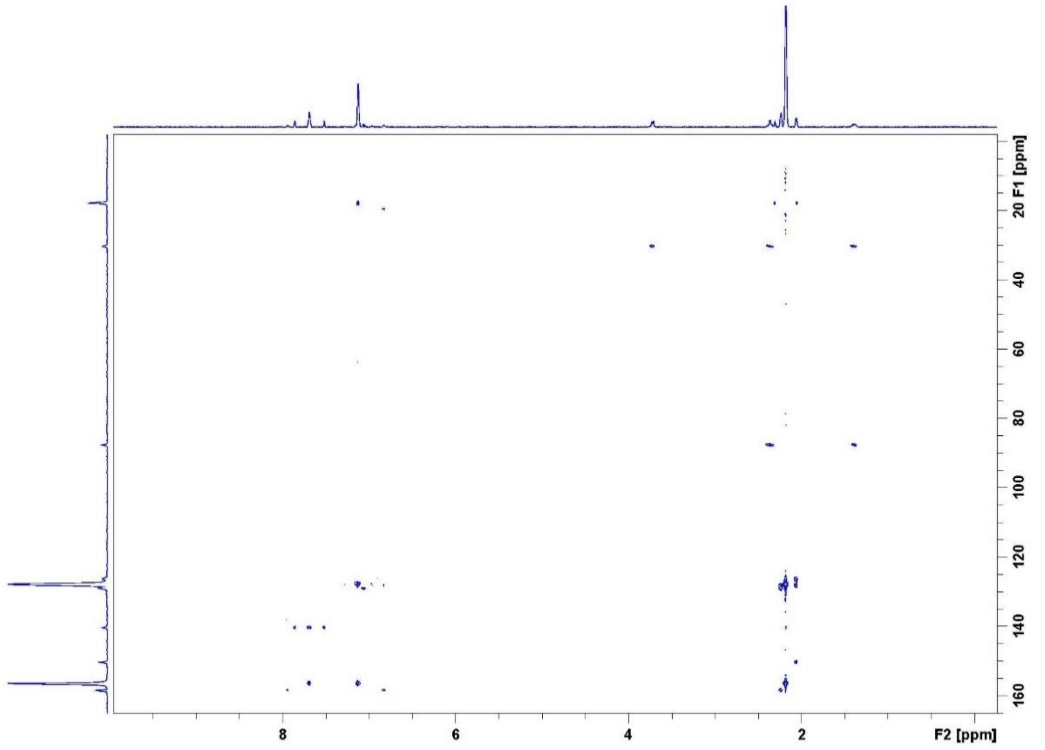

$^{DMP}$DAB–Ni–COD **2**, HR-MS

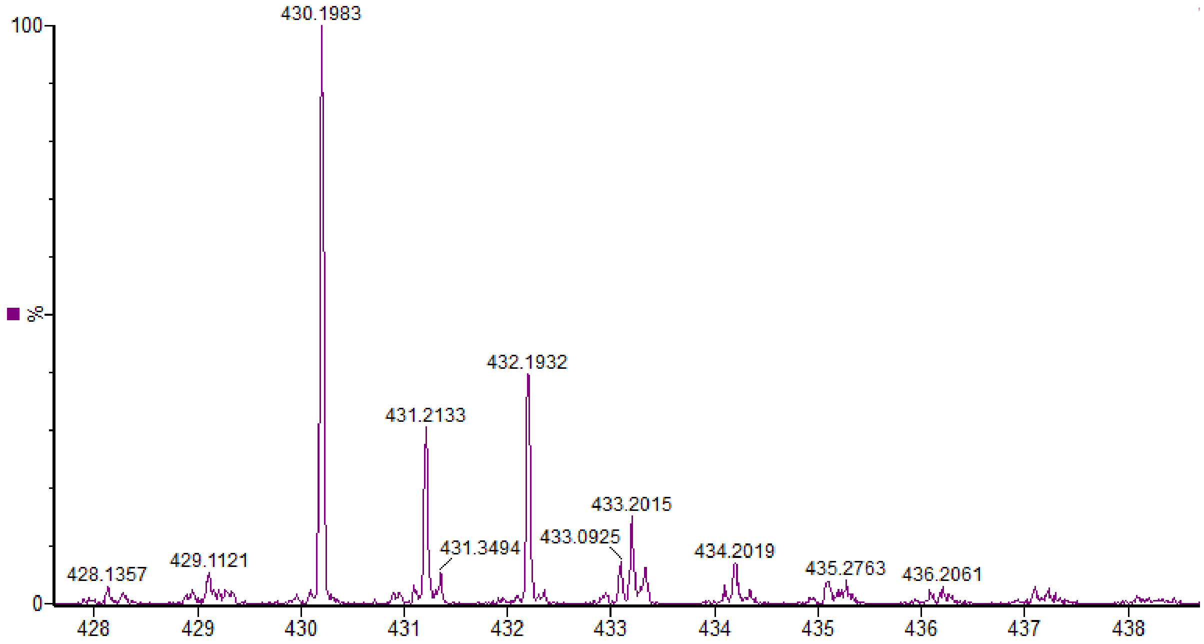


$^{DMP}$DAB–Ni–COD **2**, IR

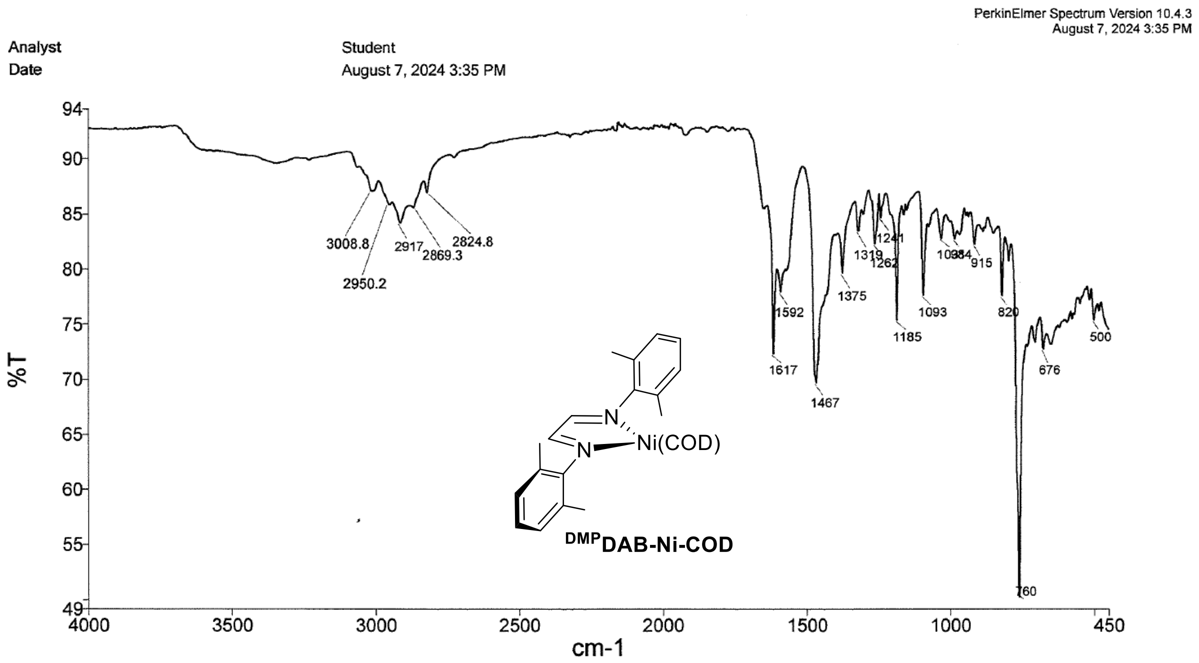

$^{DIPP}$DAB–Ni–FN **3**, $^{1}H$, 500 MHz, $C_6D_6$

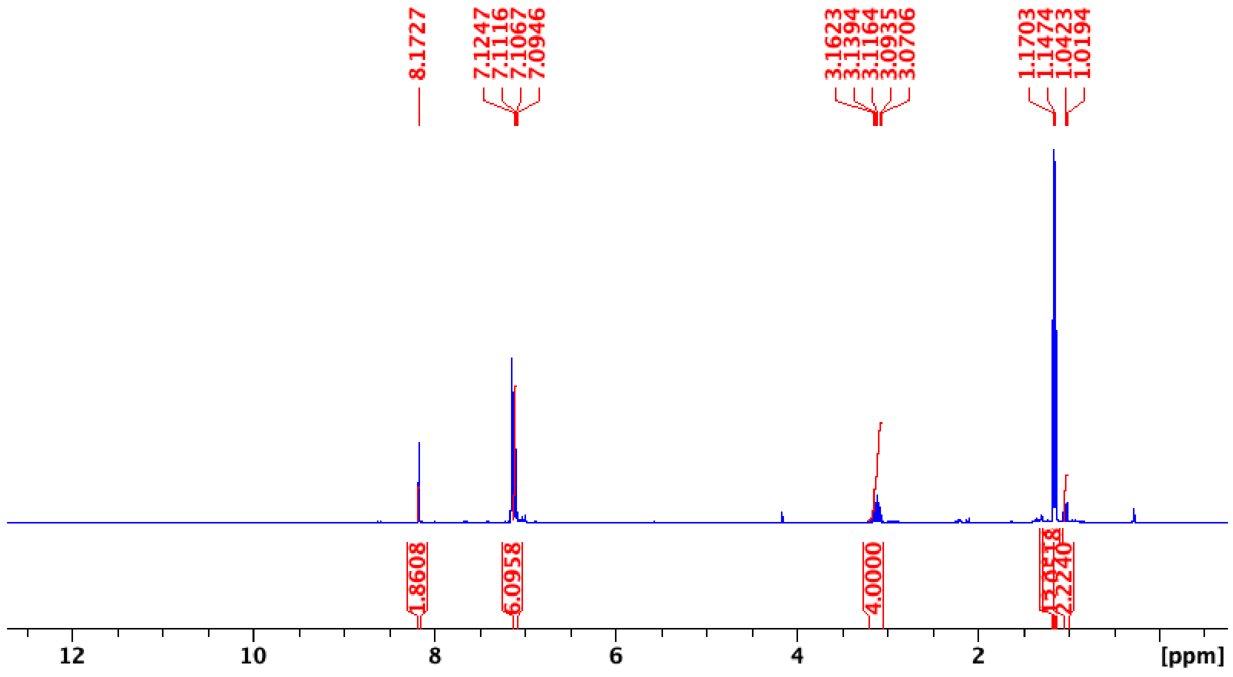


$^{DIPP}$DAB–Ni–FN **3**, $^{13}C\{^{1}H\}$, 125 MHz, $C_6D_6$

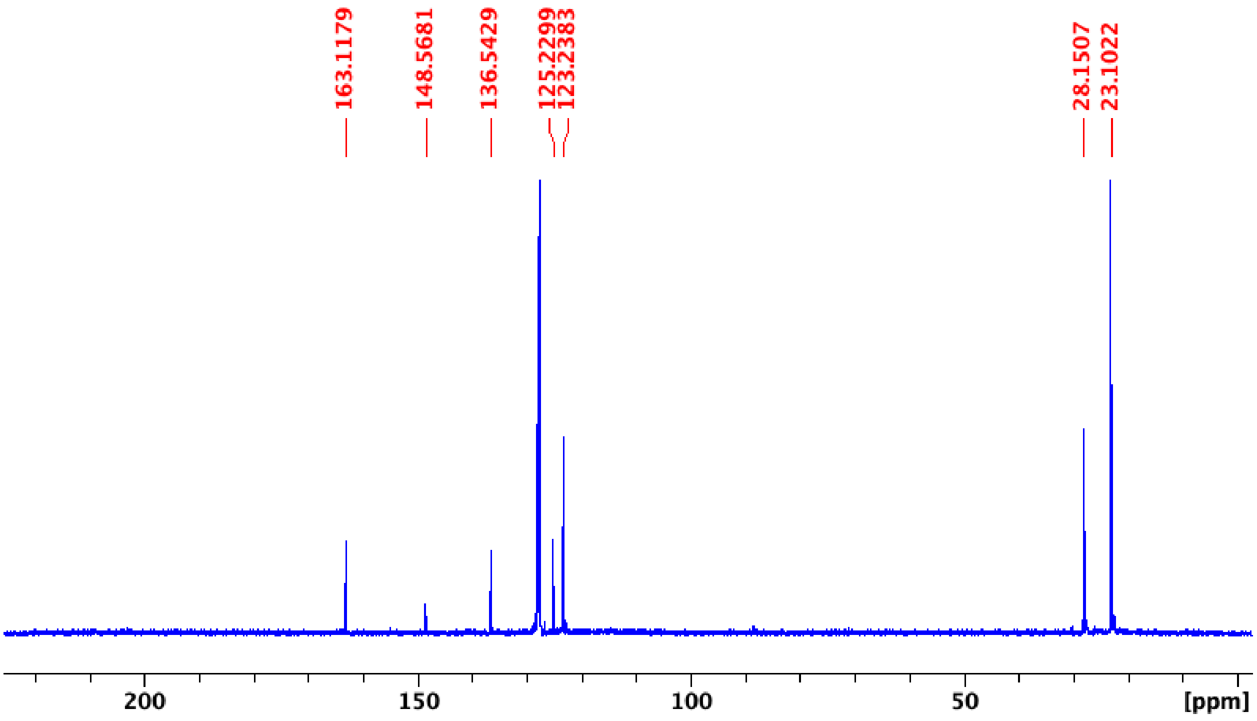

$^{DIPP}$DAB–Ni–FN **3**, $^{1}$H $^{13}$C HSQC, $C_6D_6$

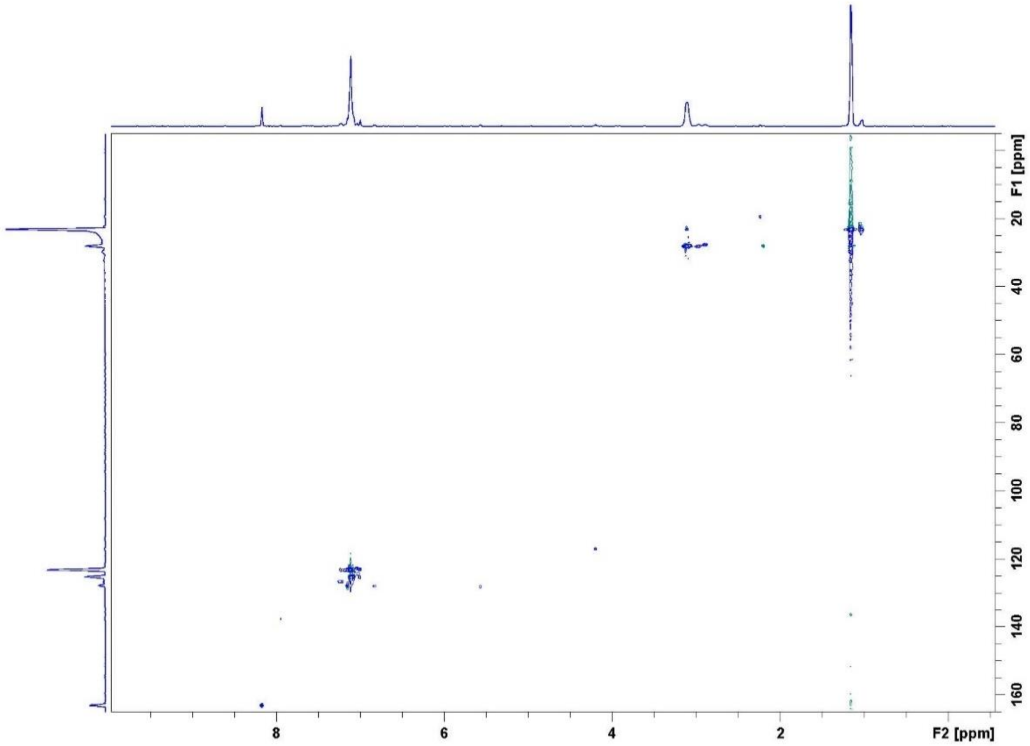


$^{DIPP}$DAB–Ni–FN **3**, $^{1}$H $^{13}$C HMBC, $C_6D_6$

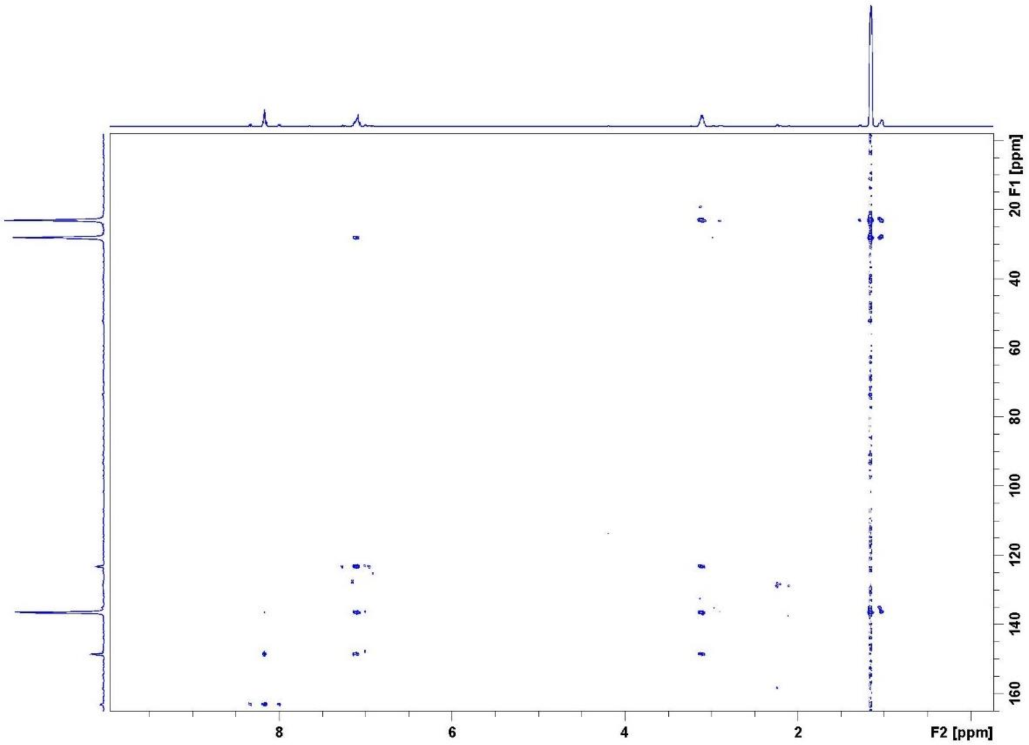

$^{DIPP}$DAB–Ni–FN **3**, IR

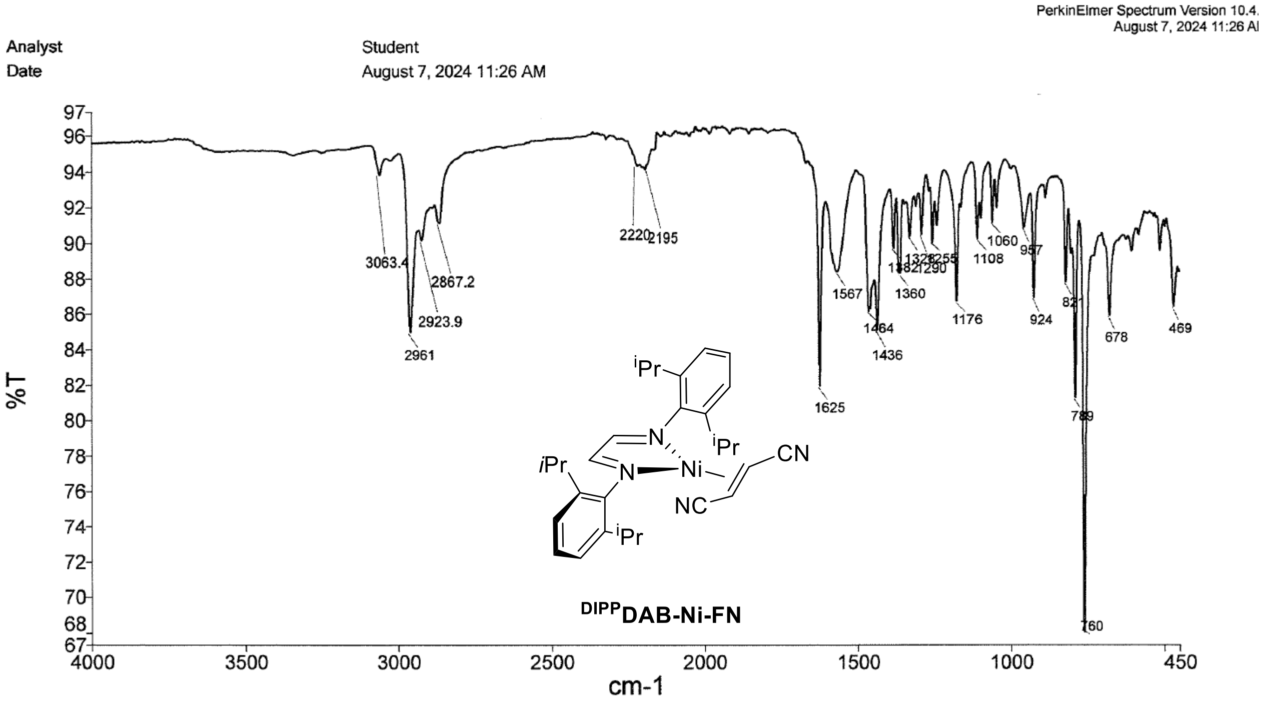

$^{DMP}$DAB–Ni–FN **4**, $^{1}$H, 500 MHz, $C_6D_6$

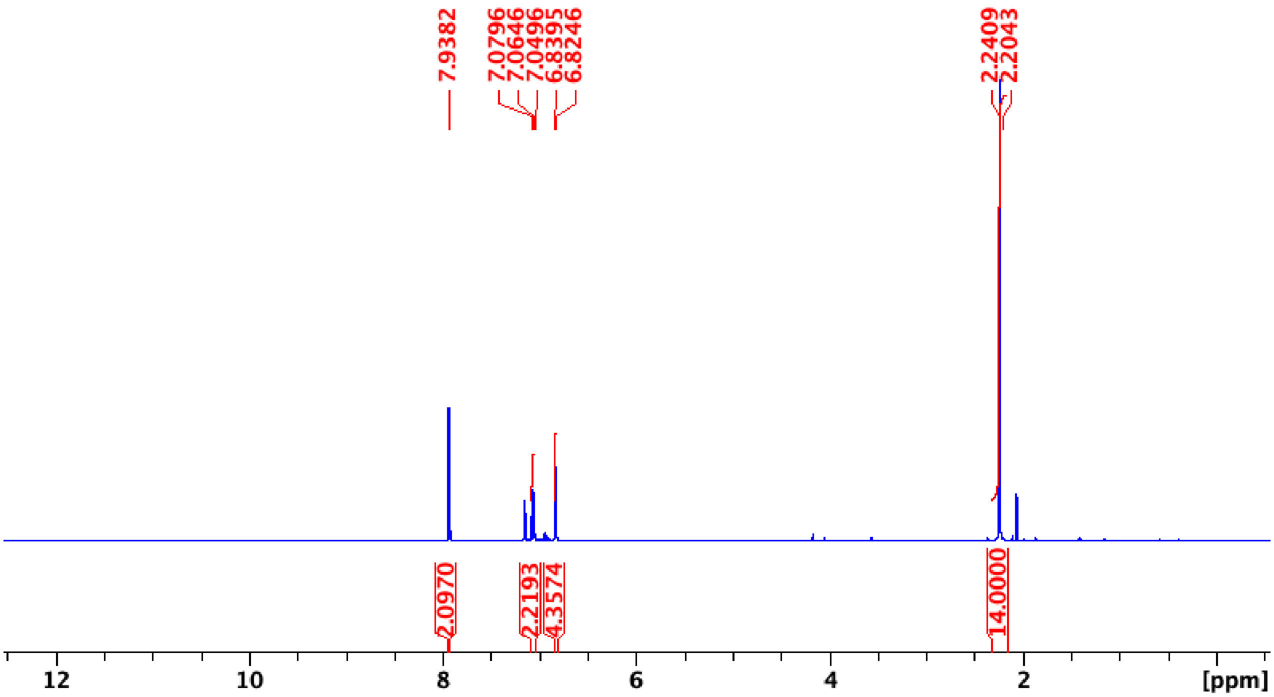


$^{DMP}$DAB–Ni–FN **4**, $^{13}C\{^{1}H\}$, 125 MHz, $C_6D_6$

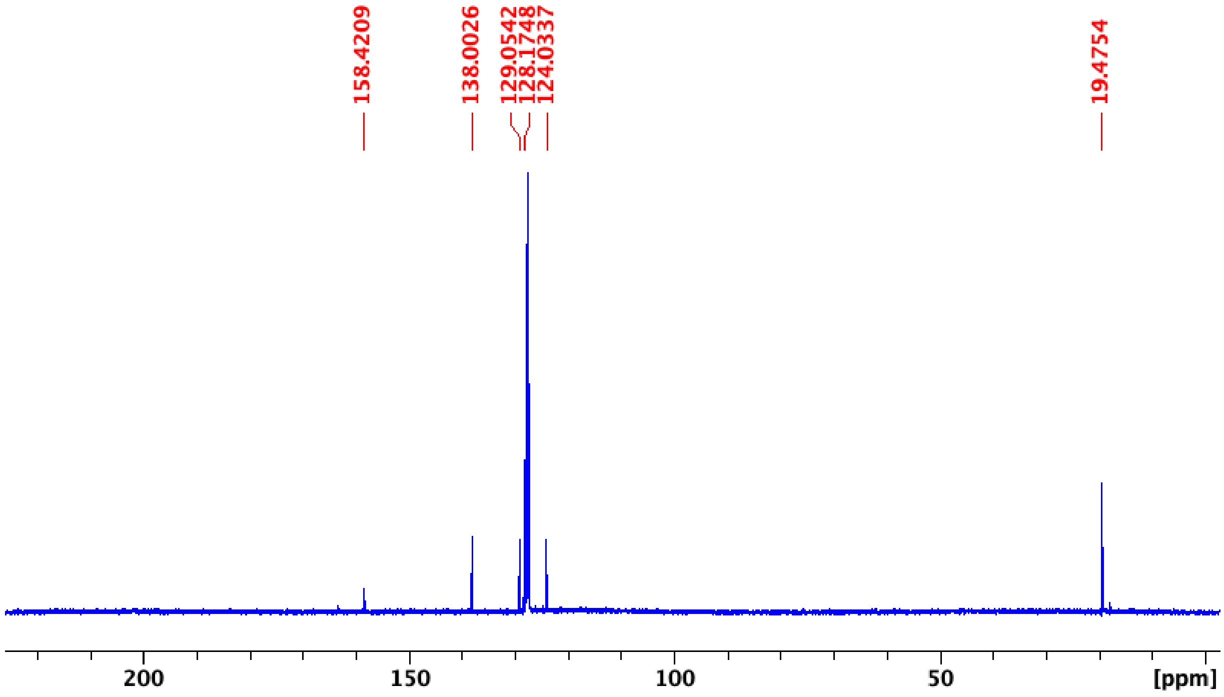

$^{DMP}$DAB–Ni–FN **4**, $^{1}$H $^{13}$C HSQC, $C_6D_6$

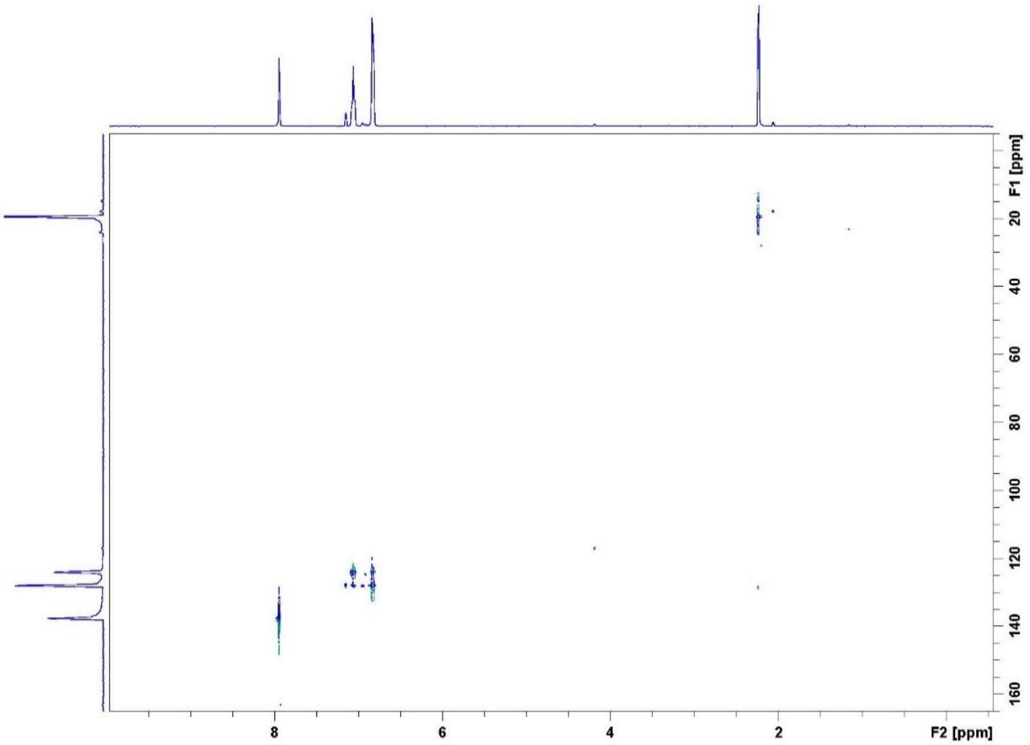


$^{DMP}$DAB–Ni–FN **4**, $^{1}$H $^{13}$C HMBC, $C_6D_6$

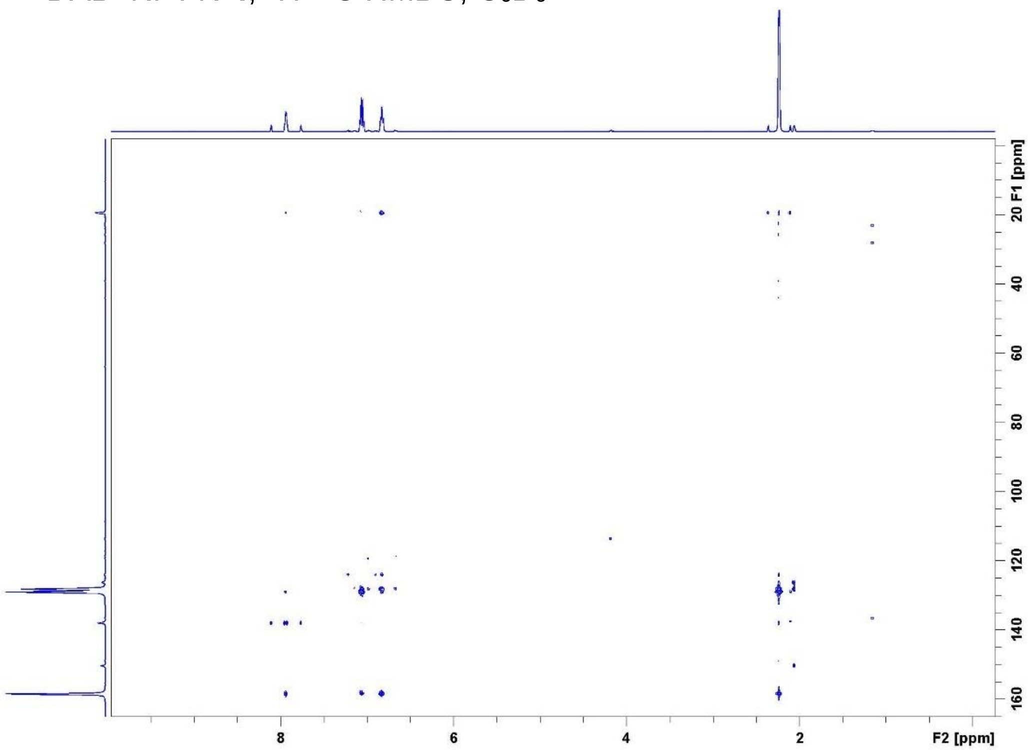

$^{DMP}$DAB–Ni–FN **4**, IR

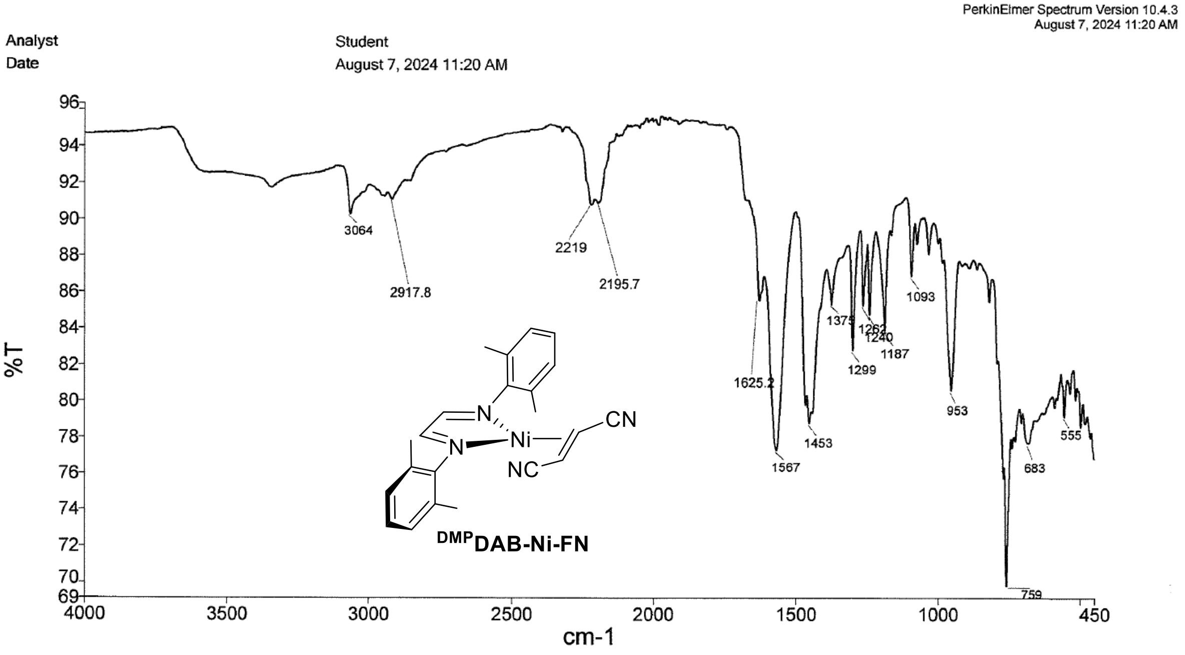

*tert*-Butyl 4-(4-fluorophenyl)-1*H*-imidazole-1-carboxylate, $^{1}H$, 300 MHz, $CDCl_3$

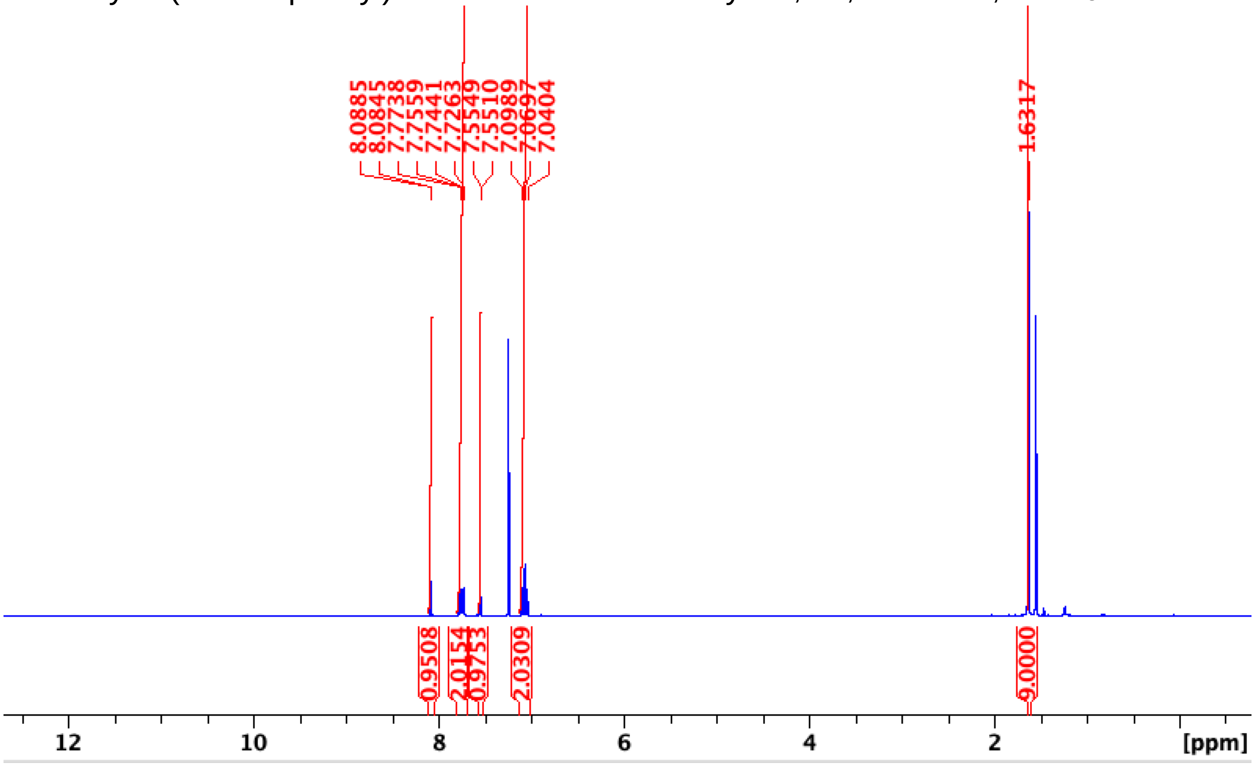


*tert*-Butyl 4-(4-fluorophenyl)-1*H*-imidazole-1-carboxylate, $^{13}C\{^{1}H\}$, 125 MHz, $CDCl_3$

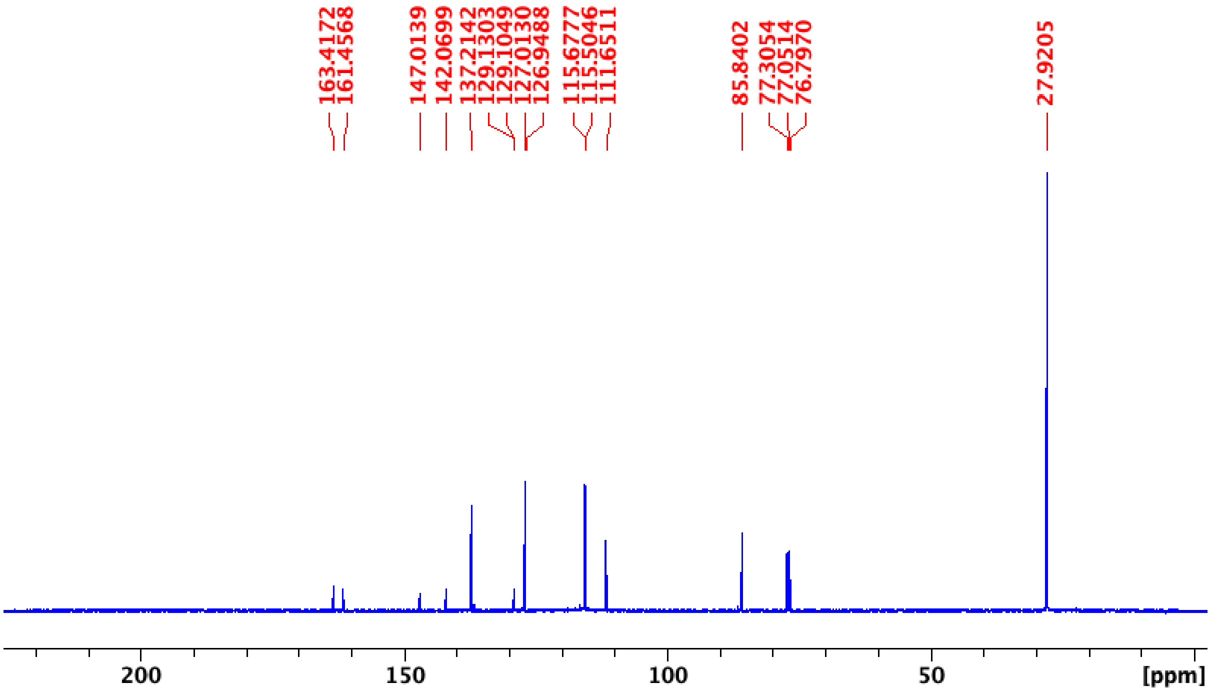

*tert*-Butyl 4-(4-fluorophenyl)-1*H*-imidazole-1-carboxylate, $^{19}F\{^1H\}$, 282 MHz, $CDCl_3$

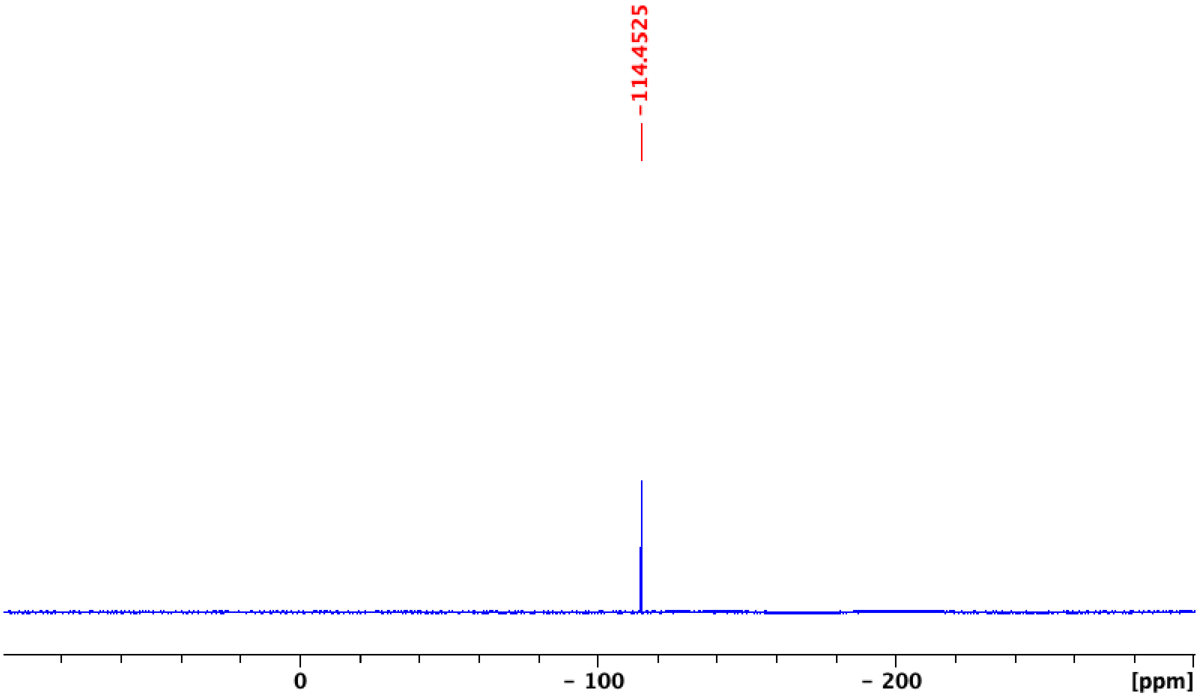


*tert*-Butyl 4-(4-fluorophenyl)-1*H*-imidazole-1-carboxylate, HR-MS

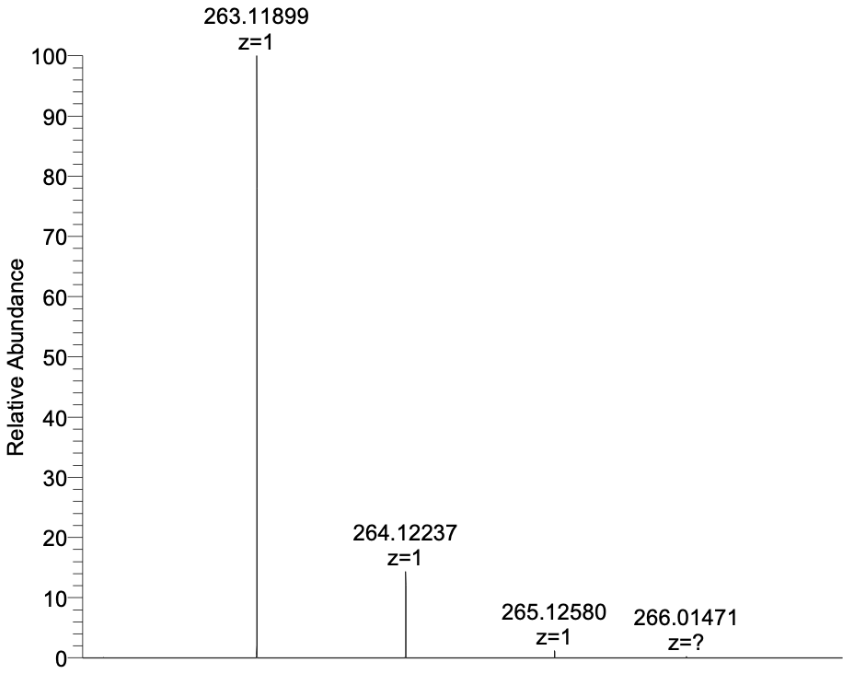

## VIII. References


[1] L. K. Johnson, C. M. Killian, M. Brookhart, *J. Am. Chem. Soc.* **1995**, *117*, 6414–6415.

[2] J. Zheng, Y. Yao, M. Li, L. Wang, X. Zhang, *J. Membr. Sci.* **2018**, *565*, 104–111.

[3] A. Liang, K. Wang, Y. Gao, B. P. Finkenauer, C. Zhu, L. Jin, L. Huang, L. Dou, *Angew. Chem. Int. Ed.* **2021**, *60*, 8337–8343.

[4] E. K. Reeves, J. N. Humke, S. R. Neufeldt, *J. Org. Chem.* **2019**, *84*, 11799–11812.

[5] M. J. Sgro, D. W. Stephan, *Dalton Trans.* **2010**, *39*, 5786–5794.

[6] M. Feuerstein, H. Doucet, M. Santelli, *J. Organomet. Chem.* **2003**, *687*, 327–336.

[7] Z.-Y. Wang, Q.-N. Ma, R.-H. Li, L.-X. Shao, *Org. Biomol. Chem.* **2013**, *11*, 7899–7906.

[8] L. Cai, X. Qian, W. Song, T. Liu, X. Tao, W. Li, X. Xie, *Tetrahedron* **2014**, *70*, 4754–4759.

[9] V. H. Tran, H.-K. Kim, *Org. Biomol. Chem.* **2022**, *20*, 2881–2888.
